# Selective synthesis of formamides, 1,2-bis(N-heterocyclic)ethanes and methylamines from cyclic amines and CO_2_/H_2_ catalyzed by an ionic liquid–Pd/C system†
†Electronic supplementary information (ESI) available: Supporting figures and tables. See DOI: 10.1039/c9sc03242h


**DOI:** 10.1039/c9sc03242h

**Published:** 2019-09-03

**Authors:** Ruipeng Li, Yanfei Zhao, Huan Wang, Junfeng Xiang, Yunyan Wu, Bo Yu, Buxing Han, Zhimin Liu

**Affiliations:** a Beijing National Laboratory for Molecular Sciences , Key Laboratory of Colloid, Interface and Thermodynamics , CAS Research , Institute of Chemistry , Chinese Academy of Sciences , Beijing 100190 , China . Email: liuzm@iccas.ac.cn ; Email: lianyi302@iccas.ac.cn; b University of Chinese Academy of Sciences , Beijing 100049 , China; c Physical Science Laboratory , Huairou National Comprehensive Science Center , China

## Abstract

The reduction of CO_2_ with amines and H_2_ generally produces *N*-formylated or *N*-methylated compounds over different catalysts. Herein, we report the selective synthesis of formamides, 1,2-bis(N-heterocyclic)ethanes, and methylamines, which is achieved over an ionic liquid (IL, *e.g.*, 1-butyl-3-methylimidazolium tetrafluoroborate, [BMIm][BF_4_])–Pd/C catalytic system. By simply varying the reaction temperature, formamides and methylamines can be selectively produced, respectively, in high yields. Interestingly, 1,2-bis(N-heterocyclic)ethanes can also be obtained *via* the McMurry reaction of the formed formamide coupled with subsequent hydrogenation. It was found that [BMIm][BF_4_] can react with formamide to form a [BMIm]^+^–formamide adduct; thus combined with Pd/C it can catalyze McMurry coupling of formamide in the presence of H_2_ to afford 1,2-bis(N-heterocyclic)ethane. Moreover, Pd/C–[BMIm][BF_4_] can further catalyze the hydrogenolysis of 1,2-bis(N-heterocyclic)ethane to access methylamine. [BMIm][BF_4_]–Pd/C was tolerant to a wide substrate scope, giving the corresponding formamides, 1,2-bis(N-heterocyclic)ethanes or methylamines in moderate to high yields. This work develops a new route to produce *N*-methylamine and opens the way to produce 1,2-bis(N-heterocyclic)ethane from cyclic amine as well.

## Abstract 2

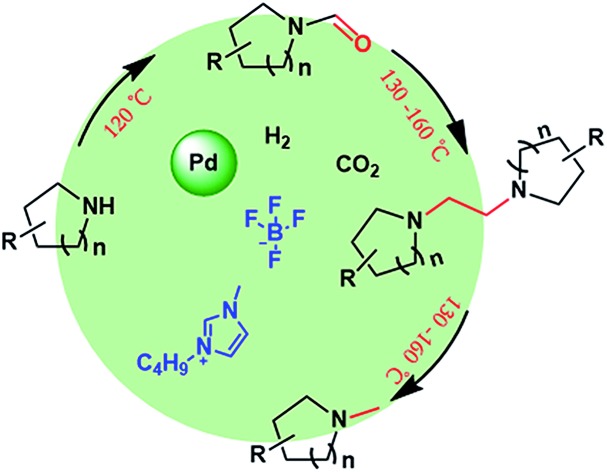
The reduction of CO_2_ with amines and H_2_ generally produces *N*-formylated or *N*-methylated compounds over different catalysts.

## Introduction

Carbon dioxide (CO_2_) is an abundant, readily available, nontoxic and renewable C1 building block, and its transformation into value-added chemicals and fuels is of great significance for green and sustainable development.^1^–^6^ The reactions of amines with CO_2_ in the presence of reductants such as hydrosilanes and H_2_ have been widely investigated, and they generally produce formamides or methylamines.^7^–^11^ For the reaction of amines with CO_2_/H_2_, the production of methylamines is more difficult than the formation of formamides,^12^–^17^ and it requires harsh reaction conditions and catalysts with very high activity.^18^–^21^ 1,2-Bis(N-heterocyclic)ethanes (*e.g.*, 1,2-bis(piperidine)ethane) are a kind of high value chemical and are generally synthesized *via* the reaction of cyclic amines with ethyl halides, suffering from production of a large amount of acid waste and complicated post-treatment.^22^,^23^ The synthesis of 1,2-bis(N-heterocyclic)ethanes from cyclic amines and CO_2_/H_2_ is a green and promising route, but this has not been realized yet.

Compared to molecular solvents (*e.g.*, water and organic solvents), ionic liquids (ILs) that are completely composed of ions have unique properties, such as a wide liquid window, very low vapor pressure, specific H-bonding between anions and cations, and so on, which make them promising media for chemical processes.^24^–^28^ In particular, they can be designed with specific functions *via* selection of suitable cations and/or anions, and have been widely applied in catalysis, showing great potential. For example, as both the solvent and catalyst, 1-ethyl-3-methylimidazolium acetate worked well for the transformation of alcohols to esters using O_2_ as an oxidant under metal-free conditions.^29^ CO_2_-philic ILs that can capture CO_2_*via* forming carbonates or carbamates have been reported to be excellent media and/or catalysts for CO_2_ transformation into value-added chemicals.^30^,^31^ 1-Butyl-3-methylimidazolium ([BMIm]^+^) chloride combined with Rh nanoparticles and ZnCl_2_, could realize the efficient reduction of heteroarenes.^32^ Carbanion-functionalized IL along with Pd(OAc)_2_ was capable of catalyzing the alkoxycarbonylation reactions of CO to benzoate products under ambient conditions.^33^ In these reactions, ILs generate synergistic effects with other active species, promoting or catalyzing the reactions.

**Scheme 1:**
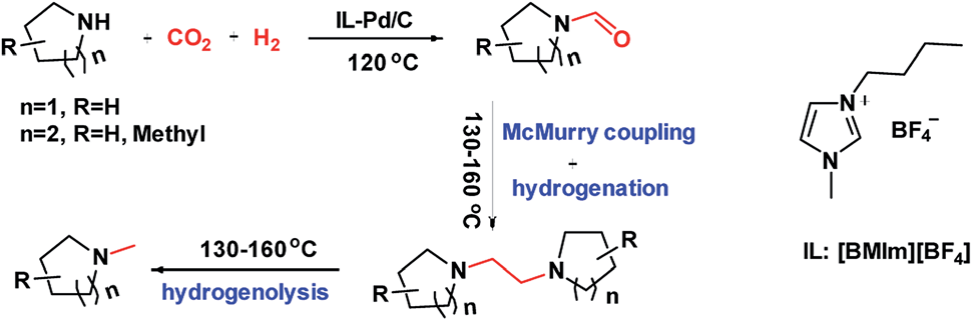
Selective reduction of CO_2_ with amines and H_2_.

Herein, we report the reduction of CO_2_ with amine and H_2_ over an IL–Pd/C catalytic system, which accomplished the selective synthesis of formamides, 1,2-bis(N-heterocyclic)ethanes and methylamines. Interestingly, the combination of Pd/C with [BMIm][BF_4_] could realize the selective production of formamides (at 120 °C) or *N*-methylamines (at 160 °C) in high yields, respectively, as illustrated in Scheme 1. Moreover, 1,2-bis(N-heterocyclic)ethanes can also be obtained *via* the McMurry reaction of the formed formamide coupled with subsequent hydrogenation. It was found that [BMIm][BF_4_] could react with formamide to form a [BMIm]^+^–formamide adduct; thus combined with Pd/C, it can catalyse McMurry coupling of formamide in the presence of H_2_ to afford 1,2-bis(N-heterocyclic)ethane and further catalyse hydrogenolysis of 1,2-bis(N-heterocyclic)ethanes to access methylamine. In addition, detailed studies indicate that the IL played multiple roles in the reactions including modifying the electronic properties of the metallic Pd particles to enhance their catalytic activity and activating the amine and the intermediate *via* strong hydrogen bonding. [BMIm][BF_4_]–Pd/C was tolerant to a wide substrate scope, giving the corresponding formamides, 1,2-bis(N-heterocyclic)ethanes or methylamines in moderate to high yields. This work develops a new route to *N*-methylamine and opens the way to produce 1,2-bis(N-heterocyclic)ethane from cyclic amine and CO_2_/H_2_.

## Results and discussion

### Preparation and characterization of the Pd/C catalyst

**Fig. 1:**
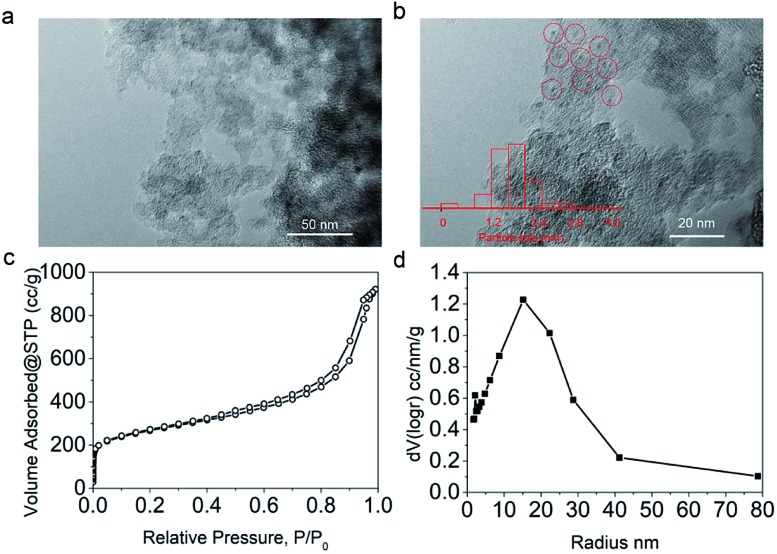
TEM (a) and HRTEM (b) images, N_2_ sorption isotherms (c) and pore size distribution (d) of the Pd/C catalyst used in this work.

To prepare the Pd/C catalyst with a loading of 5 wt%, a porous carbon support was first fabricated according to our previous report,^34^ on which Pd nanoparticles were immobilized *via* the equal volume impregnation method followed by hydrogen reduction. In the TEM images of the Pd/C catalyst (Fig. 1a and b), the dark dots were identified as Pd nanoparticles, which were uniformly distributed on the support with an average particle size around 1.7 nm. The N_2_ adsorption/desorption isotherms (Fig. 1c) indicated that the carbon support exhibited microporous and mesoporous structures with a specific surface area of 950.6 m^2^ g^–1^. The pore radius distribution curve (Fig. 1d) showed that the pore radii were centered at 15 nm according to the BJH model, a typical feature of mesoporous materials accompanied by some micropores.

### Exploration of the catalytic system for the reaction of piperidine with CO_2_/H_2_

In our initial experiments, the reaction of piperidine (**1a**) with CO_2_/H_2_ was performed using Pd/C as the catalyst to screen the catalytic system and reaction conditions for selective formation of formylpiperidine (**2a**), 1,2-bis(piperidine)ethane (**3a**) or methylpiperidine (**4a**). In the absence of CO_2_ and H_2_, no product was detected. As shown in Table 1, the solvents significantly affected the product selectivity. Molecular solvents including ethanol, tetrahydrofuran (THF) and octane exclusively afforded **2a** (Table 1, entries 1–3), while imidazolium-based ILs including [BMIm][BF_4_], [BMIm][PF_6_], [BMIm][NTf_2_] and [BMIm][Cl] could offer **2a**, **3a** and **4a** in different yields under the experimental conditions (Table 1, entries 4–9). Among these ILs, [BMIm][BF_4_] showed the best performance for the production of **4a**, achieving a yield of 90% within 9 h (Table 1, entry 5). However, only **2a** was obtained in the ILs [P_4444_][BF_4_] and [N_4444_][BF_4_] (Table 1, entries 10 and 11). These results indicated that for the imidazolium-based ILs the cations played a key role in the formation of **4a**, and the anions influenced the activity of the cations. Given that **3a** is an important chemical and it has not been accessed from **1a** and CO_2_/H_2_, we optimized the reaction conditions to obtain **3a** as the main product (Table S1†). Combined with Pd/C the mixtures of [BMIm][BF_4_] with THF or with [BMIm][Cl] could afford **3a** in yields higher than 50% at 160 °C (Table 1, entries 12 and 13). Considering that using THF as the reaction medium only **2a** was obtained, it can be deduced that [BMIm][BF_4_] and Pd/C combine to catalyze the formation of **3a** and **4a**.

**Table 1:** The reaction of piperidine with CO_2_/H_2_ over various catalytic systems^*a*^

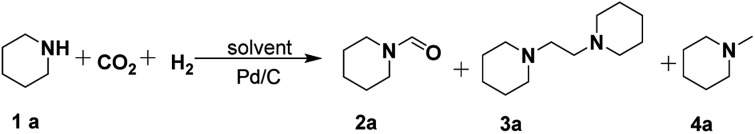				
Entry	Solvent	Yield^*e*^ (%)		
		**2a**	**3a**	**4a**
1	Ethanol	99	0	0
2	THF	99	0	0
3	Octane	99	0	0
4	[BMIm][BF_4_]	6	11	82
5^*b*^	[BMIm][BF_4_]	3	6	90
6	[HMIm][BF_4_]	17	22	49
7	[BMIm][Cl]	81	14	1
8	[BMIm][PF_6_]	46	Trace	53
9	[BMIm][NTf_2_]	67	13	18
10	[P_4444_][BF_4_]	99	0	0
11	[N_4444_][BF_4_]	99	0	0
12^*c*^	[BMIm][BF_4_] + [BMIm][Cl]	16	57	26
13^*d*^	[BMIm][BF_4_] + THF	1	77	22

^*a*^Conditions: **1a** (0.5 mmol), Pd/C (20 mg), solvent (1 mL) or IL (5 mmol), H_2_ (6 MPa), total pressure of 10 MPa, 160 °C, 6 h.

^*b*^9 h.

^*c*^H_2_ (5 MPa), total pressure of 8 MPa, [BMIm][BF_4_] (1.7 mmol), [BMIm][Cl] (3.3 mol), 9 h.

^*d*^H_2_ (5 MPa), total pressure of 8 MPa, [BMIm][BF_4_] (2.5 mmol), THF (2.5 mL), 9 h.

^*e*^Yield was determined by GC using trimethoxybenzene as the internal standard.

**Fig. 2:**
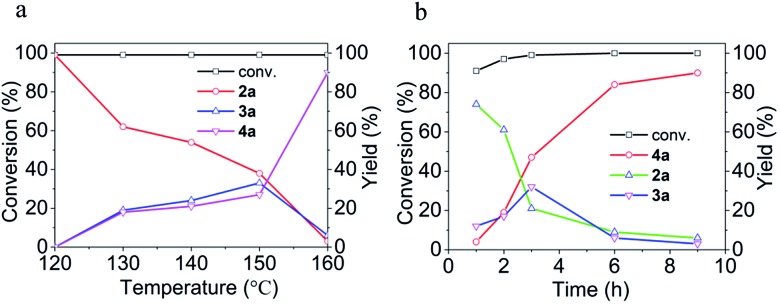
Effects of temperature (a) and reaction time (b) on the conversion of **1a** and the yields of **2a**, **3a** and **4a**. (a) 9 h. (b) 160 °C.

Since [BMIm][BF_4_]–Pd/C showed the best performance for methylation of **1a** with CO_2_/H_2_ to **4a**, it was applied to explore the effects of temperature and reaction time on the reaction. As illustrated in Fig. 2a, the reaction temperature significantly influenced the product selectivity. At 120 °C, **2a** was the sole product in an almost quantitative yield within 9 h, while both **3a** and **4a** were detected in the temperature range of 130–160 °C with the **4a** yield increasing with temperature up to 90% at 160 °C. Notably, the yield of **3a** was maintained around 20% in this temperature range, while at 160 °C it decreased significantly and **4a** became the main product. From the dependence of the **2a** and **3a** yields on the reaction time at 160 °C (Fig. 2b), it was deduced that **2a** and **3a** were finally converted into **4a** under the experimental conditions.

### Generality of the [BMIm][BF_4_]–Pd/C catalytic system

**Scheme 2:**
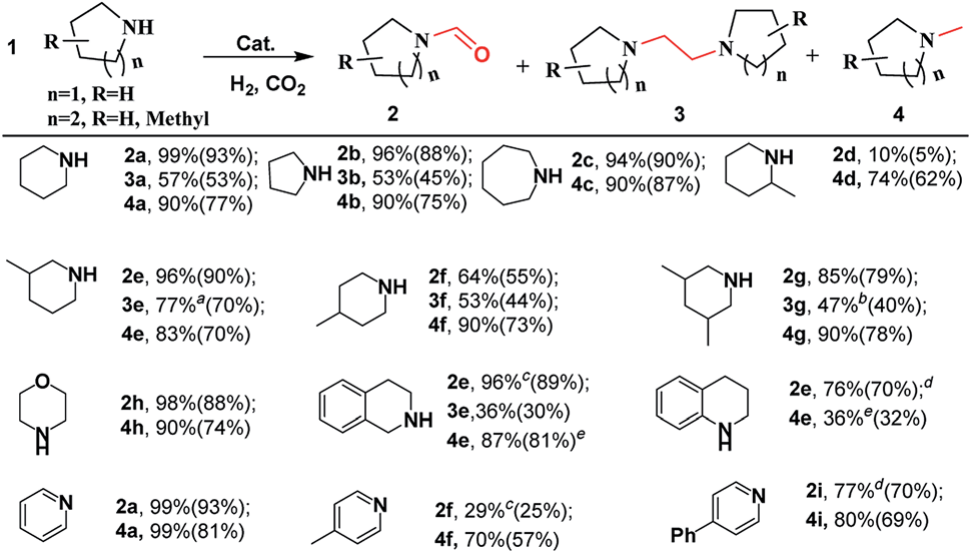
Substrate scope for selective reduction of CO_2_ with amine and H_2_ over the Pd/C catalyst. For *N*-formylation: substrate (0.5 mmol), Pd/C (20 mg), [BMIm][BF_4_] (5 mmol), H_2_ (3 MPa), total pressure of 5 MPa, 120 °C, 6 h; isolated yield in brackets. For methylation: H_2_ (6 MPa), total pressure of 10 MPa, 160 °C, 6 h; the others are the same as those for *N*-formylation. For accessing 1,2-bis(N-heterocyclic)ethanes: substrate (0.5 mmol), Pd/C (20 mg), [BMIm][BF_4_] (1.7 mmol), [BMIm][Cl] (3.3 mmol), H_2_ (5 MPa), total pressure of 8 MPa, 6 h. ^*a*^12 h. ^*b*^10 h. ^*c*^140 °C, 9 h. ^*d*^140 °C, 12 h, ^*e*^160 °C, 24 h.

To explore the generality of the [BMIm][BF_4_]–Pd/C catalytic system, it was applied in the reactions of various cyclic amines with CO_2_/H_2_. As shown in Scheme 2, this catalytic system was effective for catalyzing the reactions of the tested substrates with CO_2_/H_2_, and formylated and methylated products were selectively obtained at 120 and 160 °C, respectively. Pyrrolidine, piperidine, hexamethyleneimine, and morpholine exhibited good reactivity, affording corresponding formylated and methylated products in 90–99% yields under the optimized conditions. By using substituted piperidines like 3-methylpiperidine, 4-methylpiperidine, 2-methylpiperidine, 3,5-dimethylpiperidine, 1,2,3,4-tetrahydroisoquinoline and 1,2,3,4-tetrahydroquinoline as substrates, the reaction also proceeded smoothly and selectively furnished the desired products in moderate to high yields. However, 2-methylpiperidine was converted into the corresponding formamide (**2d**) only in 10% yield at 120 °C and 1,2,3,4-tetrahydroquinoline led to the corresponding methylamine in low yield.

After detailed screening of the reaction conditions, 1,2-bis(N-heterocyclic)ethanes could be achieved in moderate yields in most cases. The highest yield of 1,2-bis(N-heterocyclic)ethane (*e.g.*, **3e**) reached up to 77%. Interestingly, using pyridine, 4-methylpyridine, and 4-phenylpyridine as the substrates, they were first hydrogenated, and the corresponding formamides and methylamines were obtained in high yields. However, in these cases corresponding 1,2-bis(N-heterocyclic)ethanes were detected in small amounts, and the reason is unclear. In addition, [BMIm][BF_4_]–Pd/C was also effective in the reaction of chain secondary amines (*e.g.*, 1,2-diaminopropane) with CO_2_/H_2_, selectively producing formamide or methylamine in excellent yields, but it was difficult to access the corresponding 1,2-bis(amino)ethane (Scheme S1†).

### The roles of the IL

To explore the roles of the IL in the reactions, the interactions of the IL with Pd/C and with **1a**, **2a**, and **3a** were investigated. Thermogravimetric analysis on the Pd/C catalysts after adsorption of IL at 120 °C and 160 °C (Fig. S1†) indicates that the amounts of the IL adsorbed on Pd/C reached 1.28 and 0.69 wt%, respectively, and its decomposition temperature (500 °C) was much higher than that of the free IL (380 °C). These results indicate that the IL had strong interaction with Pd/C.

**Fig. 3:**
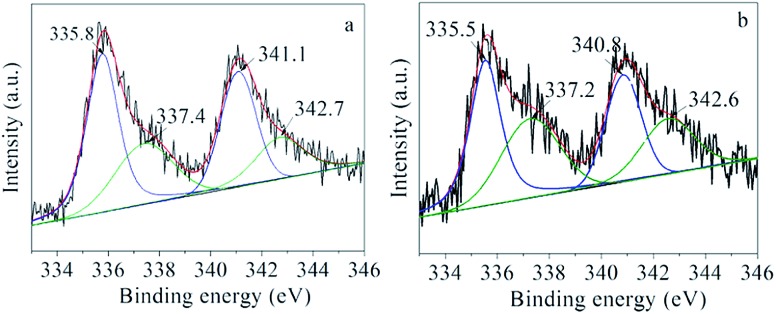
Pd 3d XPS spectra of (a) Pd/C and (b) IL–Pd/C.

The Pd/C adsorbing 1.28 wt% [BMIm][BF_4_] (denoted as IL–Pd/C) was examined by XPS. In the XPS spectrum of IL–Pd/C, the peaks at 340.8 (Pd 3d_3/2_) and 335.5 eV (Pd 3d_5/2_) attributed to Pd^0^ in Pd/C–IL exhibited a downshift of 0.3 eV compared with those of Pd/C (Fig. 3), indicating a possible electron transfer from the IL to the metallic Pd particles. This was also verified by the upshift of the N 1s peak of IL–Pd/C as compared to that of IL–C (Fig. S2,† IL–C refers to the IL adsorbed on a carbon support). The IR analysis (Fig. S3†) indicates that the adsorbed IL on Pd/C showed a red-shifted C–N stretching band of the IL cation from 1580 to 1575 cm^–1^, which is consistent with electron donation from N atoms in [BMIm]^+^ to the Pd particles. FT-EXAFS spectra also gave evidence that the IL was complexed with Pd (Fig. S4†). It is reasonable that the lone electron pair of N can affect the electronic state of the Pd particles attached to it because one N atom in the [BMIm]^+^ cation is essentially in the sp^2^ hybridization state.

**Fig. 4:**
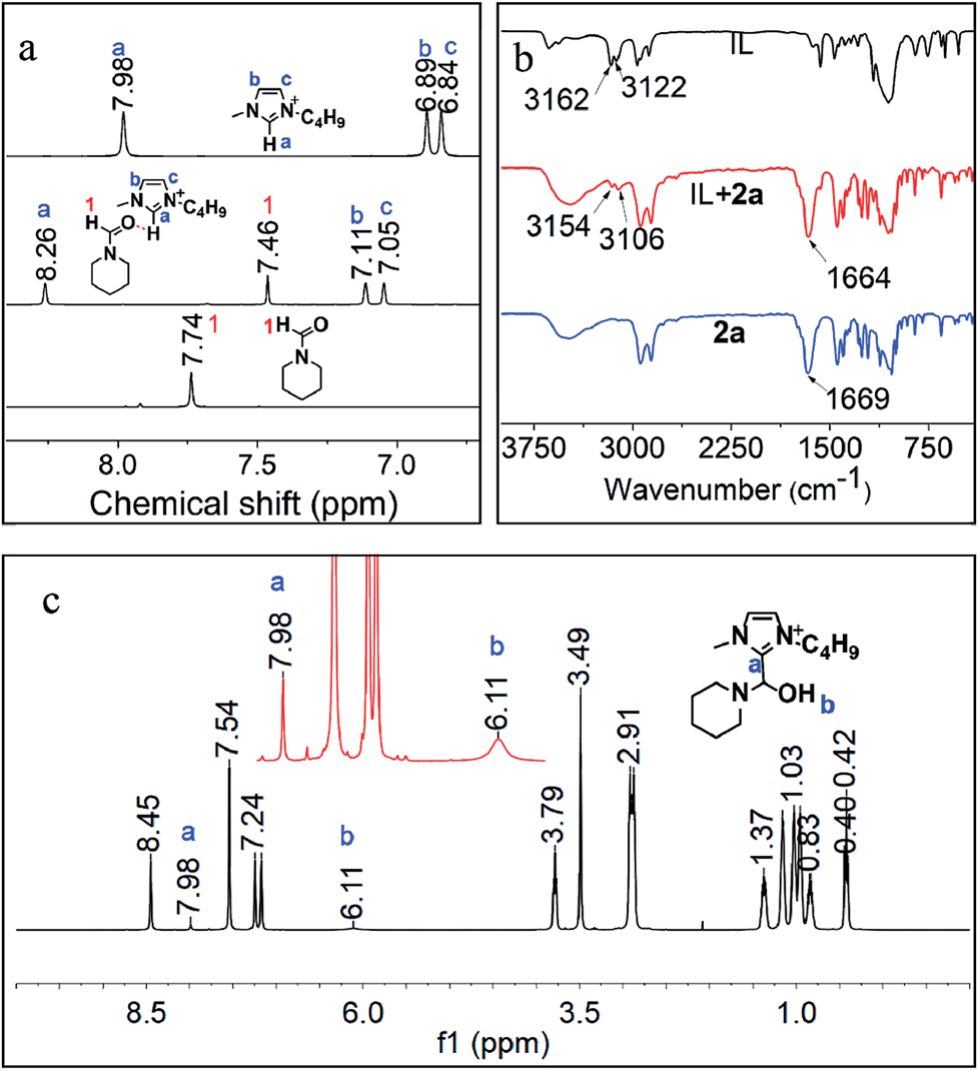
^1^H NMR (a) and FT-IR spectra (b) of [Bmim][BF_4_], **2a** and their mixture, and ^1^H NMR (c) of the mixture of IL and **2a** together with K_2_CO_3_.

Furthermore, the interactions of [BMIm][BF_4_] with CO_2_, **1a**, **2a** and **3a** were investigated *via* NMR analysis. No new signals or obvious chemical shifts were observed in the ^1^H, ^13^C, ^19^F and ^11^B NMR spectra of the mixture of [BMIm][BF_4_] and CO_2_ (Fig. S5†), indicating that this IL cannot activate CO_2_ noticeably. Given that **1a** is easily converted to a white solid, dihydropyridine-monocarboxylic acid, once exposed to CO_2_, it is supposed that CO_2_ is activated by **1a***via* forming an adduct. ^1^H, ^15^N, ^11^B NMR (Fig. S6, S7A and B†) and FTIR (Fig. S8†) analyses on the [BMIm][BF_4_]–**1a** mixture indicate that **1a** was activated by the IL *via* hydrogen bonding and electrostatic interaction. In the ^1^H NMR spectrum of the [BMIm][BF_4_]–**2a** mixture (Fig. 4a), the ^1^H signal of the formyl group in **2a** and that of the H atoms in [BMIm]^+^ shifted greatly, suggesting the strong C–H···O interaction between the acidic H(2) of [BMIm]^+^ and the O atom of the formyl group in **2a**. Specifically, the ^13^C NMR signals of the formyl group also shifted accordingly (Fig. S9†), further revealing the activation of the formyl group by the IL. In the FTIR spectrum, the absorption band at 1669 cm^–1^ attributed to the stretching vibration of C=O of **1a** shifted to 1664 cm^–1^ upon **1a** interacting with [BMIm][BF_4_], and the peaks at 3162 and 3122 cm^–1^ belonging to the stretching vibration of C–H from the imidazole ring of [BMIm]^+^ shifted accordingly. The FTIR analysis gave evidence of interaction between **1a** and the IL, also supporting the NMR results (Fig. 4b). As reported, reactive carbenes can be generated at the C(2) position of [BMIm]^+^ under mild basic conditions.^35^ In this work, the basic reaction environment provided by **1a** or **4a** may be favorable for the formation of carbenes from [BMIm]^+^, which may further react with **2a** to form an intermediate. To confirm this hypothesis, a mixture of IL and **2a** together with K_2_CO_3_ was examined. Based on ^1^H NMR and ^1^H–^1^H correlation spectroscopy (COSY) analysis (Fig. 4c and S10†), an intermediate from [BMIm][BF_4_] and **2a** (denoted as [BMIm–OH–**2a**][BF_4_]) was detected (Fig. 4c), which was also confirmed by the detection of {[BMIm–OH–**2a**][BF_4_][BMIm](**2a**)}^+^ (*m*/*z* = 591.4) by electrospray-ionization mass spectrometry (ESI-MS) (Fig. S11†). The above results indicate that the IL can activate **2a** to form a [BMIm]^+^–formamide adduct, which may be favorable for the formation of **3a** and **4a**. In addition, **3a** can also be activated by the IL, as supported by NMR analysis with the obvious changes of chemical shifts as it mixed with the IL in the presence of **2a** (Fig. S12†), which can explain why the IL promotes the hydrogenolysis of **3a**.

### Possible reaction pathway

To gain deep insight into the reaction pathway of the formation of **3a** and **4a**, control experiments were performed. Previous reports revealed that **2a** could be catalytically converted to **4a***via* hydrogenation under appropriate conditions.^36^ However, in this work taking **2a** as the substrate to react with CO_2_/H_2_ or only with H_2_ over Pd/C in [BMIm][BF_4_], no **4a** was detectable (Scheme S2A and B†). This indicates that Pd/C could not catalyze the direct hydrogenation of **2a** to **4a** in [BMIm][BF_4_], which excludes the pathway of **2a** reduction with H_2_, and implies that **2a** undergoes transformation to **3a** to form **4a**.

**Scheme 3:**
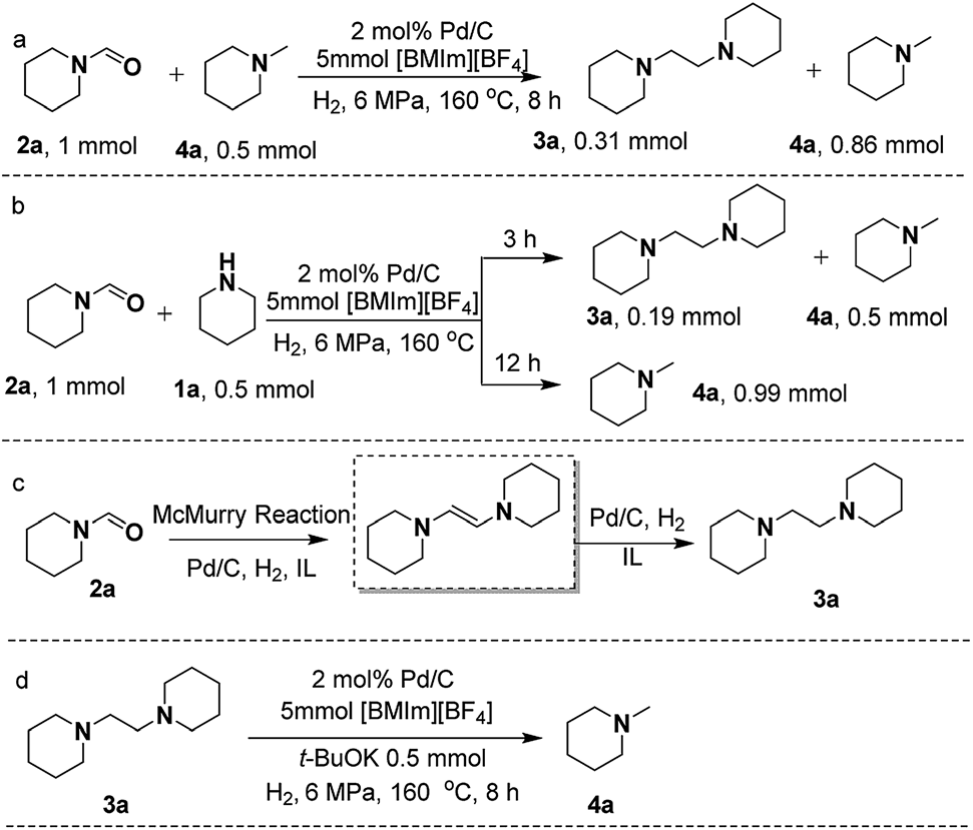
Control experiments. Conversion of **2a** to **3a** in the presence of **4a** (a). Conversion of **2a** to **3a** and/or **4a** in the presence of **1a** for different reaction times (b). The possible reaction pathway from **2a** to **3a** (c). Conversion of **3a** to **4a** in the presence of *t*-BuOK (d).

As is known, McMurry coupling is a reductive reaction, in which two ketone or aldehyde groups are coupled to form an alkene using a titanium chloride compound (*e.g.*, titanium(iv) chloride) and a reducing agent (*e.g.*, zinc) in the presence of a base.^37^ In the process of **1a** reacting with CO_2_/H_2_, the reaction system is always kept in a basic environment due to the presence of the amine feedstock or the formed methylamine, which might facilitate the occurrence of the McMurry reaction of the formed formamide. To verify this, we treated **2a** in the presence of **1a** or **4a** over Pd/C in [BMIm][BF_4_] (Scheme 3a and b), and **3a** was excitingly obtained in appreciable yields in both cases, different from the results shown in Scheme S2A and B.† This suggests that the McMurry reaction of **2a** may occur to produce 1,2-bis(piperidine)ethylene although it was not detected in the reaction process, because it can be rapidly hydrogenated to **3a** catalyzed by Pd/C (Scheme 3c). Notably, on prolonging the reaction time to 12 h, both **2a** and **3a** disappeared, and **4a** became the sole product (Scheme 3b). This indicates that **3a** was further converted into **4a***via* hydrogenolysis under the experimental conditions, which was confirmed by the reaction of **3a** with H_2_ over Pd/C in [BMIm][BF_4_] containing *t*-BuOK to produce **4a** (Scheme 3d). Generally, the C–N bond breaks more easily than the C–C bond. However, in this work the C–C bond rather than the C–N bond in **3a** was broken. To give a reasonable explanation for this, Gaussian calculation was performed, which showed that the acidic hydrogen atom H(2) of [BMIm]^+^ could interact with an N atom of **3a**, causing the length of the C–C bond in **3a** to become longer (Fig. S13†). This may be responsible for the cleavage of the C–C bond in **3a** to give **4a**. [BMIm][BF_4_] played a crucial role in the hydrogenolysis of **3a** to **4a**, deduced from the fact that without the IL the hydrogenolysis of **3a** did not occur even in the presence of the base *t*-BuOK (Scheme S2C†).

To identify possible intermediates in the reaction process, the reaction solution was analyzed by means of electrospray ionization mass spectrometry (ESI-MS.) As shown in Fig. S14,† {H[BMIm][BF_4_](**1a**)}^+^ (*m*/*z* = 312.2), {H[BMIm][BF_4_](**3a**)}^+^ (*m*/*z* = 423.3), {[BMIm][*N*-(*N*-piperidinoglycolyl)piperidine](**4a**)}^+^ (*m*/*z* = 464.4), {[BMIm-[*N*-(*N*-piperidinoglycolyl)piperidine](**2a**)}^+^ (*m*/*z* = 476.4), {[BMIm–OH–**2a**][BF_4_][BMIm](**2a**)}^+^ (*m*/*z* = 591.4) and {([BMIm][BF_4_])_2_[BF_4_][*N*-(*N*-piperidinoglycolyl)piperidine]}^–^ (*m*/*z* = 765.4) were detected, suggesting that under the experimental conditions, [BMIm–OH–**2a**][BF_4_] and *N*-(*N*-piperidinoglycolyl)piperidine were the key intermediates and [BMIm][BF_4_] played a vital role in activating and stabilizing them.

**Scheme 4:**
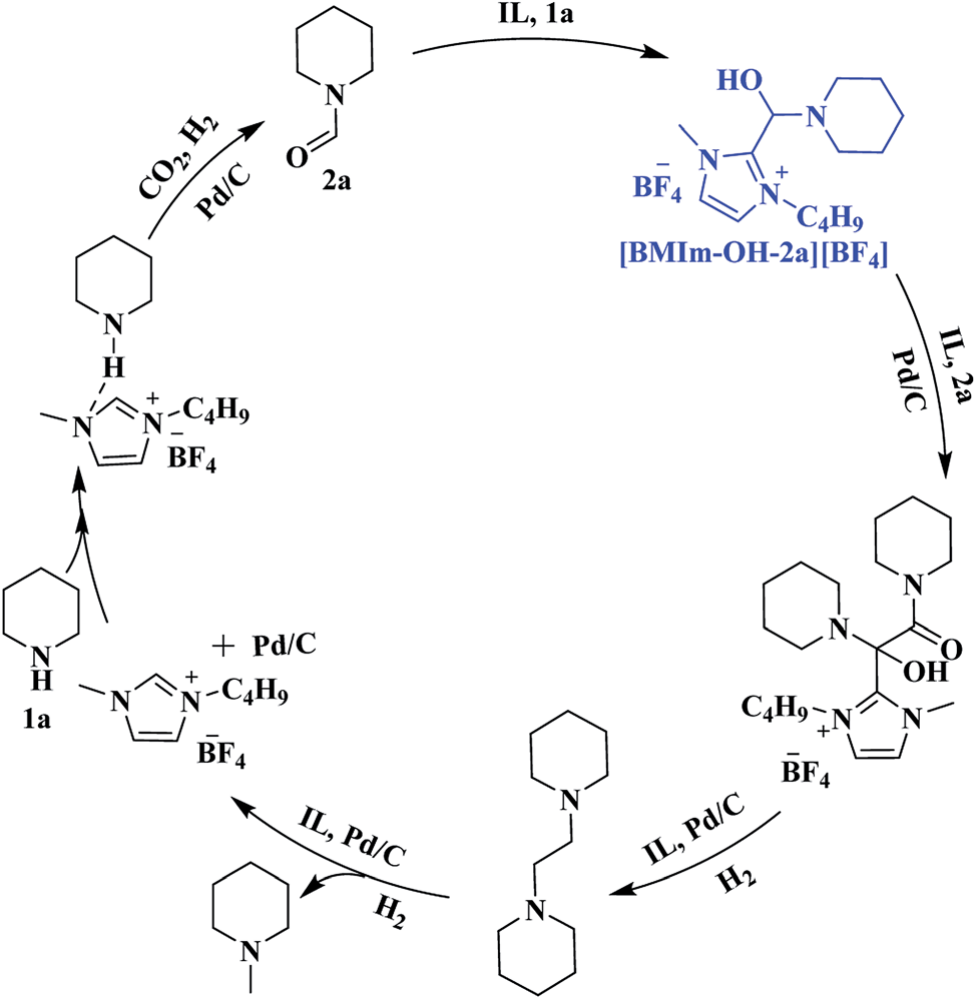
Plausible pathways for the formation of **2a**, **3a** and **4a**.

Based on the experimental results, a possible pathway is proposed, as shown in Scheme 4. Initially, **1a** is activated by the IL *via* hydrogen bonding and electrostatic interaction and undergoes formylation with CO_2_/H_2_ to yield **2a** over Pd/C. Subsequently, an intermediate [BMIm–OH–**2a**][BF_4_] is formed from **2a** and [BMIm]^+^ in the presence of **1a**, and the McMurry reaction proceeds through coupling of [BMIm–OH–**2a**][BF_4_] with **2a** to produce *N*-(*N*-piperidinoglycolyl)piperidine, followed by hydrogenation to form **3a**. Finally, hydrogenolysis of **3a** results in the formation of **4a**. Notably, this is the first time that the McMurry reaction of formamide over Pd/C using H_2_ as a reductant has been realized, very different from the traditional routes.

## Conclusions

In summary, selective reduction of CO_2_ with cyclic amines and H_2_ was realized over the [BMIm][BF_4_]–Pd/C catalytic system, and 1,2-bis(N-heterocyclic)ethanes were obtained *via* the McMurry reaction of formamide coupled with subsequent hydrogenation. [BMIm][BF_4_] displayed multiple functions including improving the catalytic activity of Pd particles and activating the amine substrate and the formed formamide intermediate. [BMIm][BF_4_]–Pd/C was tolerant to a wide substrate scope, giving the corresponding formamides, 1,2-bis(N-heterocyclic)ethanes or methylamines in moderate to high yields. We believe that these findings provide insights into achieving cooperativity between the IL and metal catalysts.

## Experimental

### General procedures for the reaction of N-containing compounds with CO_2_/H_2_

All reactions were performed in a stainless steel autoclave equipped with a Teflon tube (16 mL inner volume) and a magnetic stirrer. In a typical experiment, piperidine (0.5 mmol), [BMIm][BF_4_] (5 mmol) and Pd/C (20 mg) were successively loaded into the autoclave under an N_2_ atmosphere, and then the autoclave was sealed. H_2_ (6 MPa) and CO_2_ were charged successively into the reactor until the total pressure reached 10 MPa at room temperature. The autoclave was moved to a heating furnace at 433 K. After the desired reaction time, the reactor was cooled down in ice water and the gas inside was vented slowly. Trimethoxybenzene (internal standard) and diethyl ether were added to the reaction solution, stirred vigorously and centrifuged. The upper liquid was extracted for analysis.

### Product analysis

Liquid samples were analysed using a gas chromatograph (Agilent 4890D) equipped with an ultra-inert capillary column (19091S-433UI HP-5 ms). NMR spectra were collected using a coaxial insert NMR tube with d^6^-DMSO in the internal tube as a reference, recorded using Bruker 400 HD (^1^H NMR, ^11^B NMR and ^13^C NMR spectra) and Bruker 600 instruments (^15^N NMR and ^19^F NMR spectra and the ^1^H–^1^H correlation spectrum (^1^H–^1^H COSY). ^11^B NMR and ^19^F NMR spectra were obtained using the same equipment in succession. ^1^H NMR data recorded in d^6^-DMSO were listed with the residual DMSO at 2.50 ppm, while ^13^C NMR data were listed with the residual DMSO at 39.51 ppm; ^1^H NMR data in d-CDCl_3_ were listed with the residual CHCl_3_ at 7.26 ppm, while ^13^C NMR data were listed with the residual CHCl_3_ at 77.17 ppm. FT-IR spectra were recorded using a Bruker Tensor-27 instrument, scanning from 400 to 4000 cm^–1^. Transmission electron microscopy (TEM) images were obtained using a JEOL-2100F. X-ray photoelectron spectroscopy (XPS) was performed on an ESCALab220i-XL electron spectrometer from VG Scientific at a pressure of 3 × 10^–9^ mbar using 300 W Al-Kα radiation. The binding energies (BEs) were referenced to the C 1s line at 284.8 eV from adventitious carbon. X-ray absorption data at the Pd K-edge of the samples were recorded at room temperature in fluorescence mode with a silicon drift fluorescence detector at beam line BL14W1 of the Shanghai Synchrotron Radiation Facility (SSRF), China. The electron storage ring was operated at 3.5 GeV. Data processing was performed using the program ATHENA. The electrospray ionization mass spectrometry (ESI-MS) data (in both negative and positive modes) were collected using a Bruker 9.4T Solarix instrument.

## Conflicts of interest

There are no conflicts to declare.

## Supplementary Material

Supplementary information
