# Supplementary material for: Selective synthesis of formamides, 1,2-bis(N-heterocyclic)ethanes and methylamines from cyclic amines and CO2/H2 catalyzed by an ionic liquid–Pd/C system
Source: Chem Sci. 2019 Sep 3;10(42):9822–8. doi: 10.1039/c9sc03242h (PMC6977556; doi:10.1039/c9sc03242h)
Supplement: Supplementary file 1 [file SC-010-C9SC03242H-s001.pdf]

Electronic Supplementary Material (ESI) for Chemical Science.
This journal is © The Royal Society of Chemistry 2019

Supplementary Information for

Selective Synthesis of Formamides, 1,2-Bis(N-heterocyclic)ethanes and Methylamines from Cyclic Amines and CO_2/H_2 Catalyzed by Ionic Liquid-Pd/C system

Ruipeng Li[a,b], Yanfei Zhao[a,], Huan Wang[a,b], Junfeng Xiang[a], Yunyan Wu[a,b], Bo Yu[a], Buxing Han[a,b,c]& Zhimin Liu[a,b,c*,]*

[a]Beijing National Laboratory for Molecular Sciences, Key Laboratory of Colloid, Interface and Thermodynamics, CAS Research, Institute of Chemistry, Chinese Academy of Sciences, Beijing 100190, China. [b]University of Chinese Academy of Sciences, Beijing 100049, China. [c]Physical Science Laboratory, Huairou National Comprehensive Science Center.

E-mail: liuzm@iccas.ac.cn, lianyi302@iccas.ac.cn

Materials. 1-Butyl-3-methylimidazolium chloride ([BMIm][Cl]), 1-butyl-3-methylimidazolium tetrafluoroborate ([BMIm][BF_4]), 1-butyl-3-methylimidazolium hexafluorophosphate ([BMIm][PF_6]), 1-butyl-3-methylimidazolium bis((trifluoromethyl)sulfonyl)imide ([BMIm][NTf_2]), and 1-hexyl-3-methylimidazolium tetrafluoroborate ([HMIm][BF_4]) were purchased from Lanzhou Institute of Chemical Physics, Chinese Academy of Sciences. CO_2 and H_2 was supplied by Beijing Analytical Instrument Factory with a purity of 99.995%. And various substrates of N-containing substrates were obtained commercially from J&K Scientific Ltd. and Innochem Science & Technology Co., Ltd. respectively. The other chemicals were purchased from Beijing Chemical Reagent Company. All chemicals were of analytical grade and used as received. "IL" in this word represents [BMIm][BF_4] if there were no special explanation.

General procedure for IL adsorption on Pd/C. The adsorption of IL on Pd/C was performed under the above experimental conditions. After the adsorption, the mixture was centrifuged, washed with ethanol for several times, and then dried under vacuum at 60 ^{o}C overnight. The amount of IL adsorbed at different reaction temperature was determined by TG analysis. TG measurements were performed on a thermal analyzer (PerkinElmer TGA 4000) in N_2 with a heating rate of 10 ^{o}C /min.

Characterization. The XPS data were processed using XPSPEAK41. The Pd 3d core level XPS pattern was fitted according to asymmetric peaks with 3:2 peak area ratio and 5.3 eV peak separation. The work-up of raw XAFS data to k-space and Fourier transformed R-space involved energy calibration, background subtraction, and μ0 fitting. All data was first processed using a consistent methodology of background subtraction, conversion to k-space. Then, k2-weighting data in the k space ranging from 3.12 to 12.2 $Å^{-1}$ for Pd was Fourier transformed (FT) to R space.

DFT calculation. Gaussian calculation was performed to verify that the length of C-C bond in **3a** to become longer. This may be responsible for the cleavage of C-C bond in **3a** to give **4a**. All calculations were performed with the Gaussian 09 package. Geometry optimizations and frequency calculations were carried out at the B3LYP/6-311+G(d,p) level at 298.15 K.

Table S1. Reaction conditions screening [a].

Entry	Temperature/ ^{o}C	Solvent/Additive	Yield (%) [e]		
			**2a**	**3a**	**4a**
1	130	[BMIm][BF_4]	62	18	19
2	140	[BMIm][BF_4]	54	21	24
3	150	[BMIm][BF_4]	38	27	33
4	160	[BMIm][BF_4]	11	6	82
5 [b]	160	[BMIm][BF_4] + CsF	27	23	45
6 [b]	160	[BMIm][BF_4] + K_2CO_3	26	22	51
7 [c]	160	[BMIm][BF_4] + Ethanol	99	0	0
8 [c]	160	[BMIm][BF_4] + Octane	6	37	56
9 [d]	160	[BMIm][BF_4] + [BMIm][Cl] (1:2)	16	53	30
10 [d]	160	[BMIm][BF_4] + [BMIm][Cl] (1:1)	28	41	30

[a]Reaction conditions: substrate (0.5 mmol), Pd/C (20 mg), [BMIm][BF_4] (5 mmol), H_2 (3 MPa), total pressure of 10 MPa, 160 °C, 6 h. [b]Base (0.1 mmol). [c]IL (2.5 mmol), solvent (2.5 mL) H_2 (3 MPa), total pressure of 8 MPa, 3 h. [d]Substrate (0.5 mmol), Pd/C (20 mg), the total amount of [BMIm][BF_4] and [BMIm][Cl] was 5 mmol, while the ratio in brackets was molar ratio, H_2 (5 MPa), total pressure of 8 MPa, 4 h. [e]Yield was determined by GC using trimethoxybenzene as internal standard.

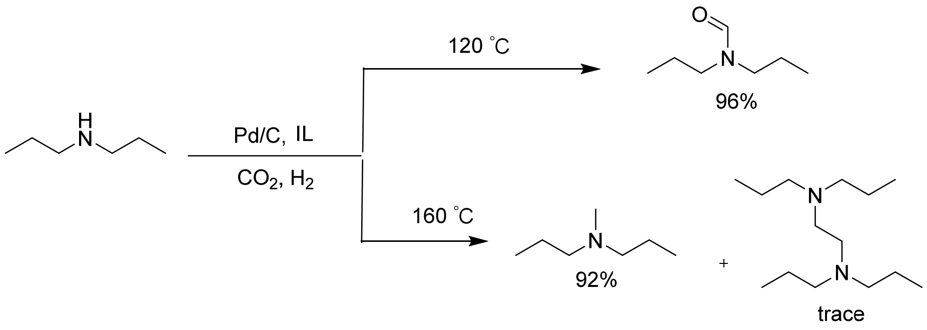

Scheme S1. Reactions of dipropylamine with CO_2 and H_2 at 120 °C and 160 °C.

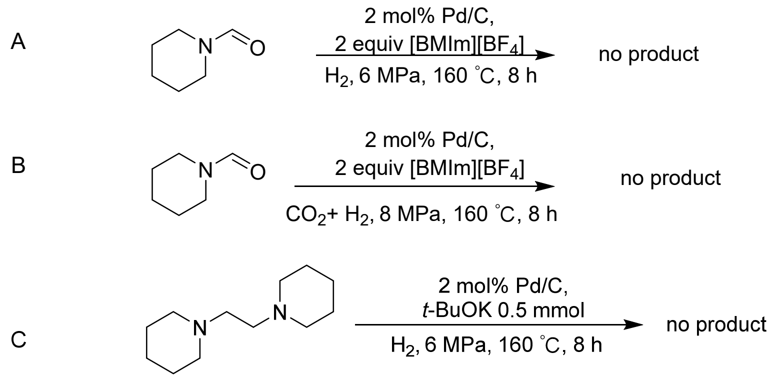

Scheme S2. Control experiments: (A) **2a** was treated with H_2. (B) **2a** was treated with H_2 and CO_2. (C) **3a** was treated with H_2 in the presence of base *t*-BuOK without IL.

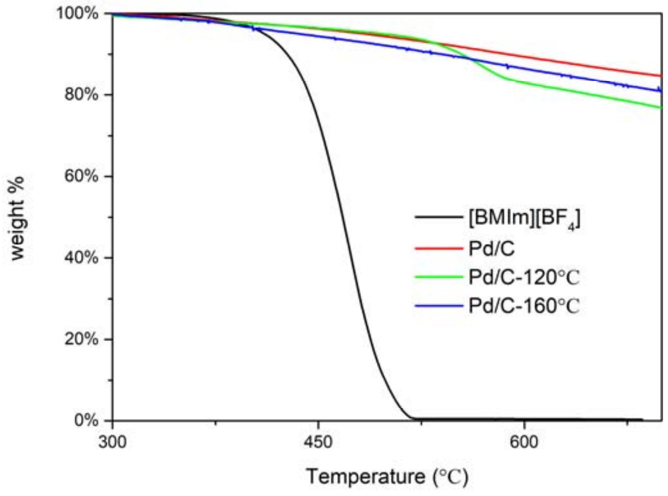

Figure S1. TG plots of Pd/C and IL-Pd/C with different absorption capacity.

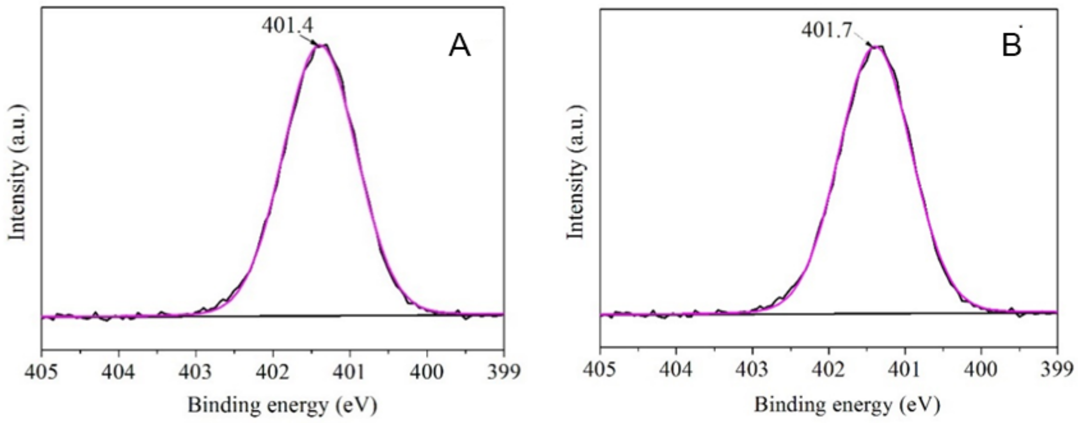

Figure S2. N1s XPS spectra of (a) IL-C and (b) IL-Pd/C.

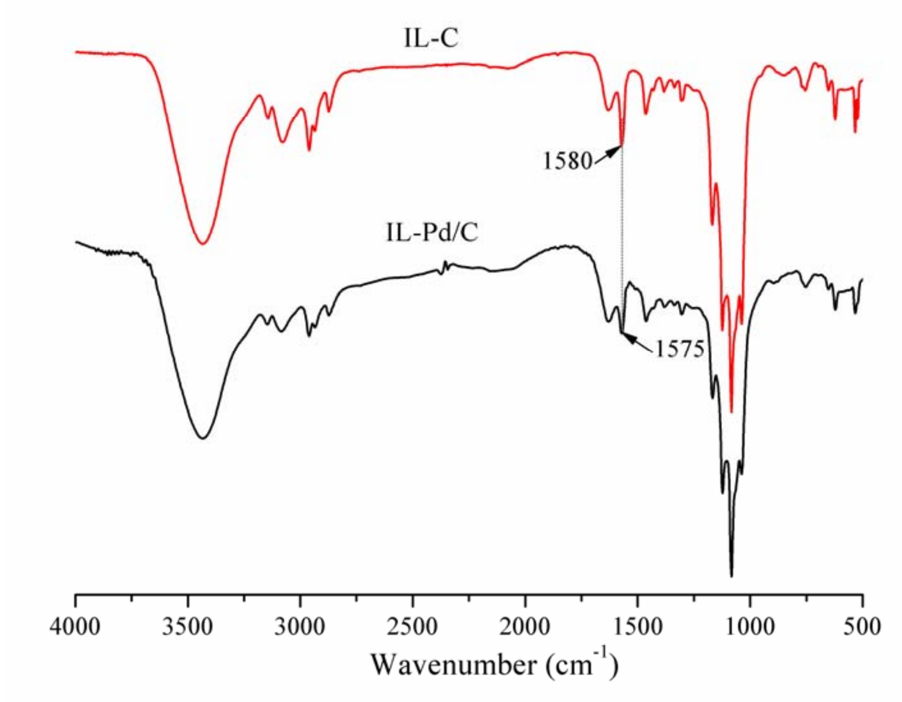

Figure S3. FTIR spectra of IL-C and IL-Pd/C.

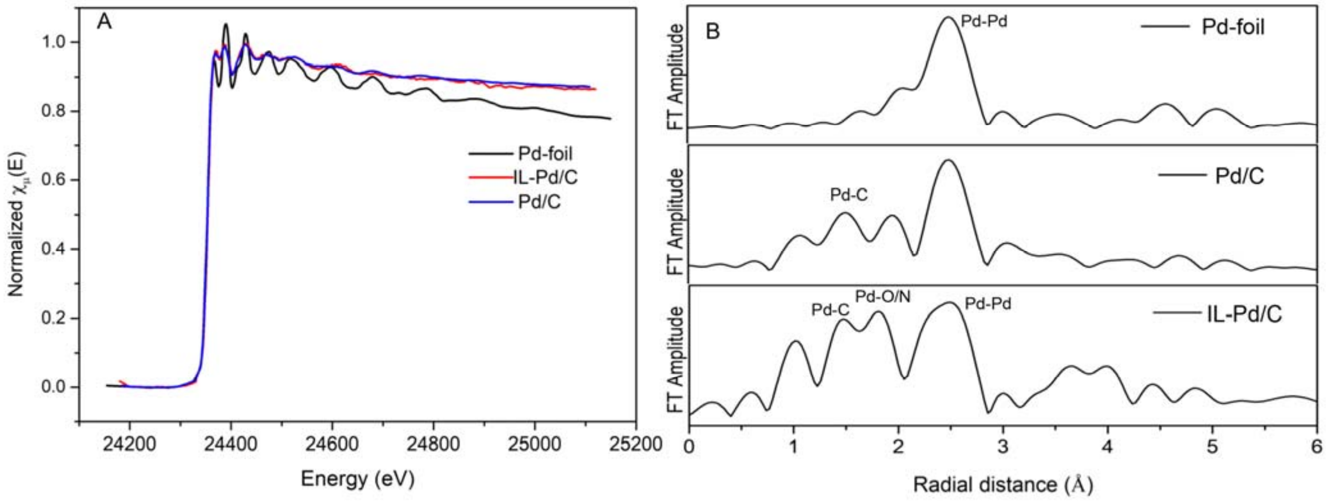

Figure S4. Normalized Pd-K-edge spectra of Pd-foil, Pd/C and Pd/C with $[BMIm][BF_4]$ (a). Fourier transforms $FT(k^3\chi\mu(K))$ for Pd-foil, Pd/C and IL-Pd/C.

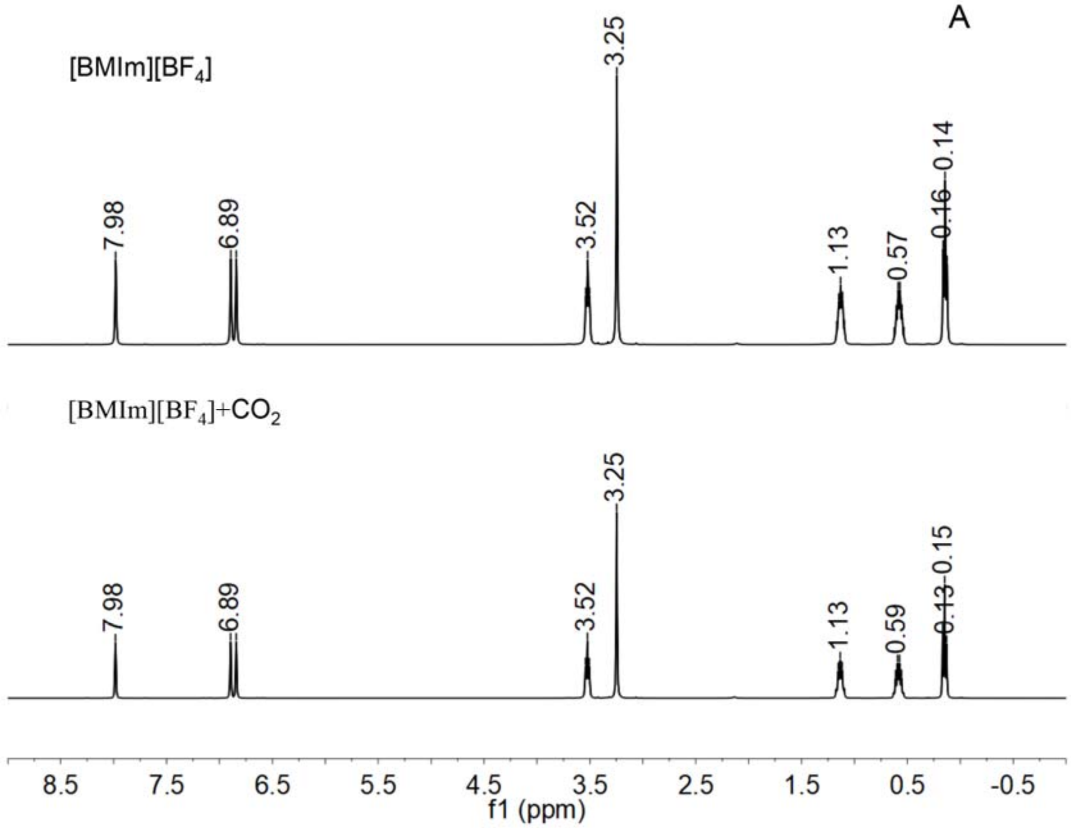

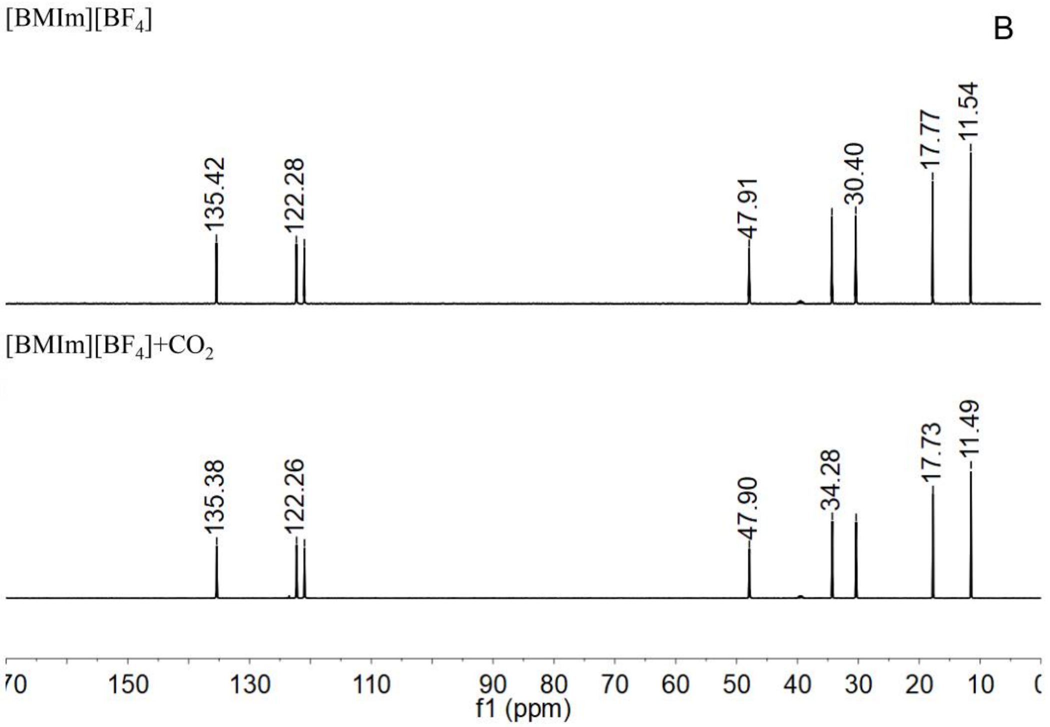

B
[BMIm][BF4]
135.42
122.28
47.91
30.40
17.77
11.54
[BMIm][BF4]+CO2
135.38
122.26
47.90
34.28
17.73
11.49
150
130
110
90
80
70
60
50
40
30
20
10
f1 (ppm)

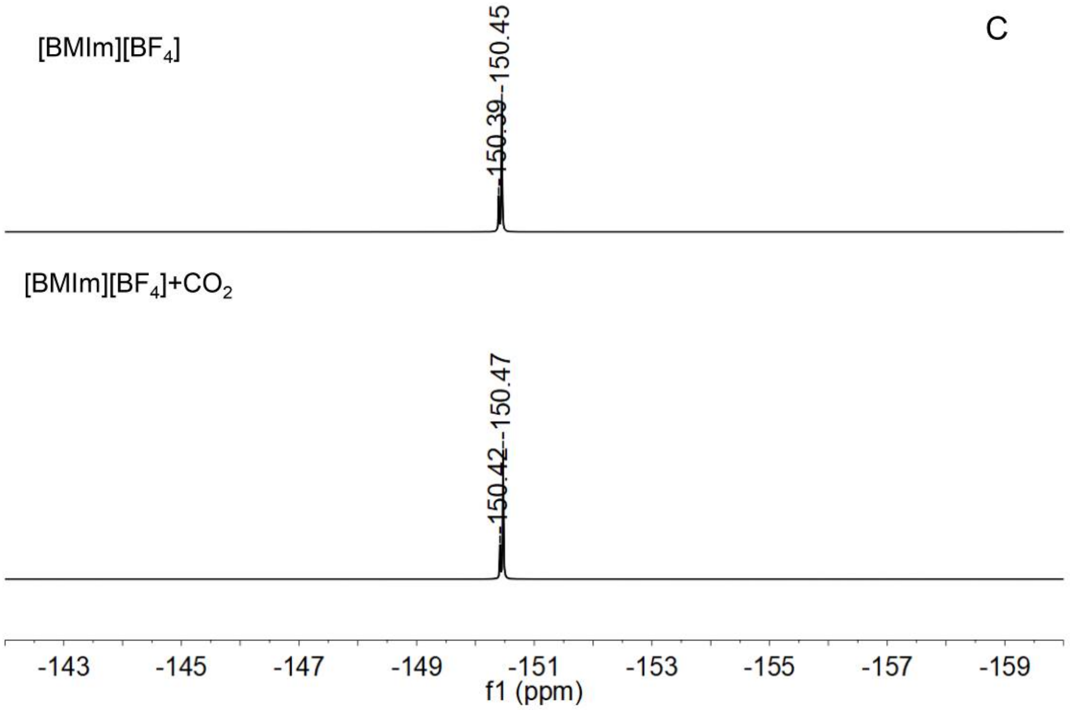

C
[BMIm][BF4]
150.45
150.39
[BMIm][BF4]+CO2
150.47
150.42
-143
-145
-147
-149
-151
-153
-155
-157
-159
f1 (ppm)

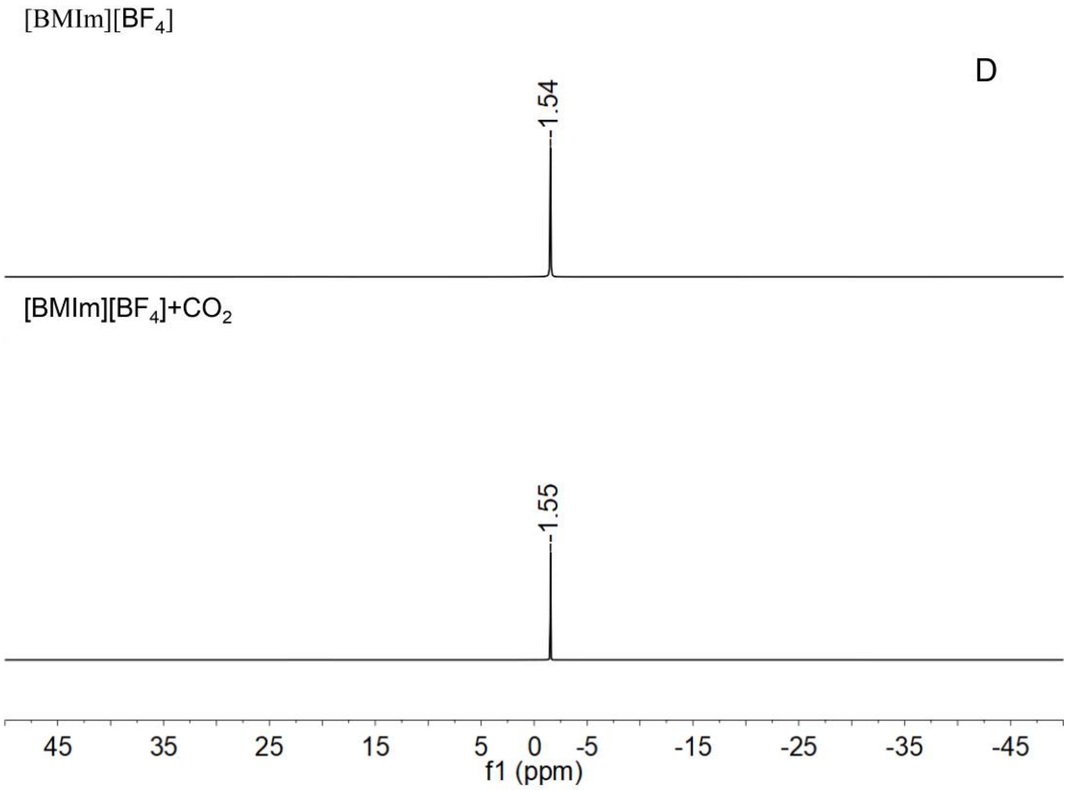

Figure S5. ^{1}H, ^{13}C, ^{19}F and ^{11}B NMR spectra of [BMIm][BF_4] before and after exposing to CO_2.

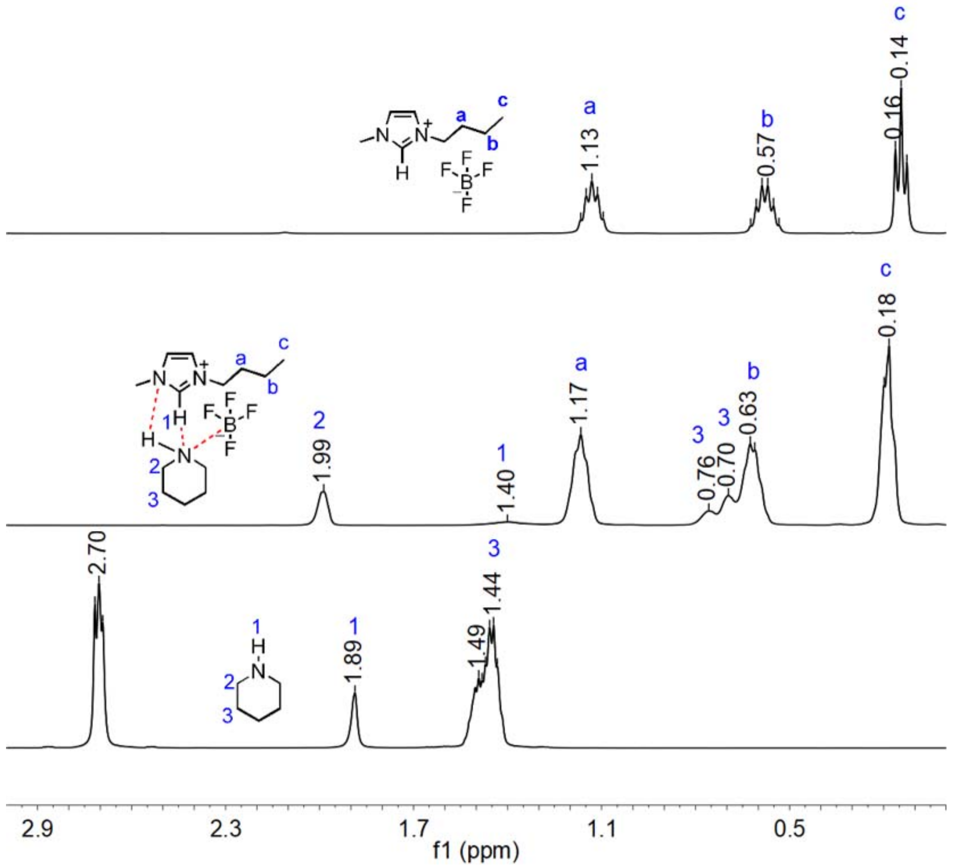

Figure S6. ^{1}H NMR spectra of [BMIm][BF_4], **1a** and their mixture.

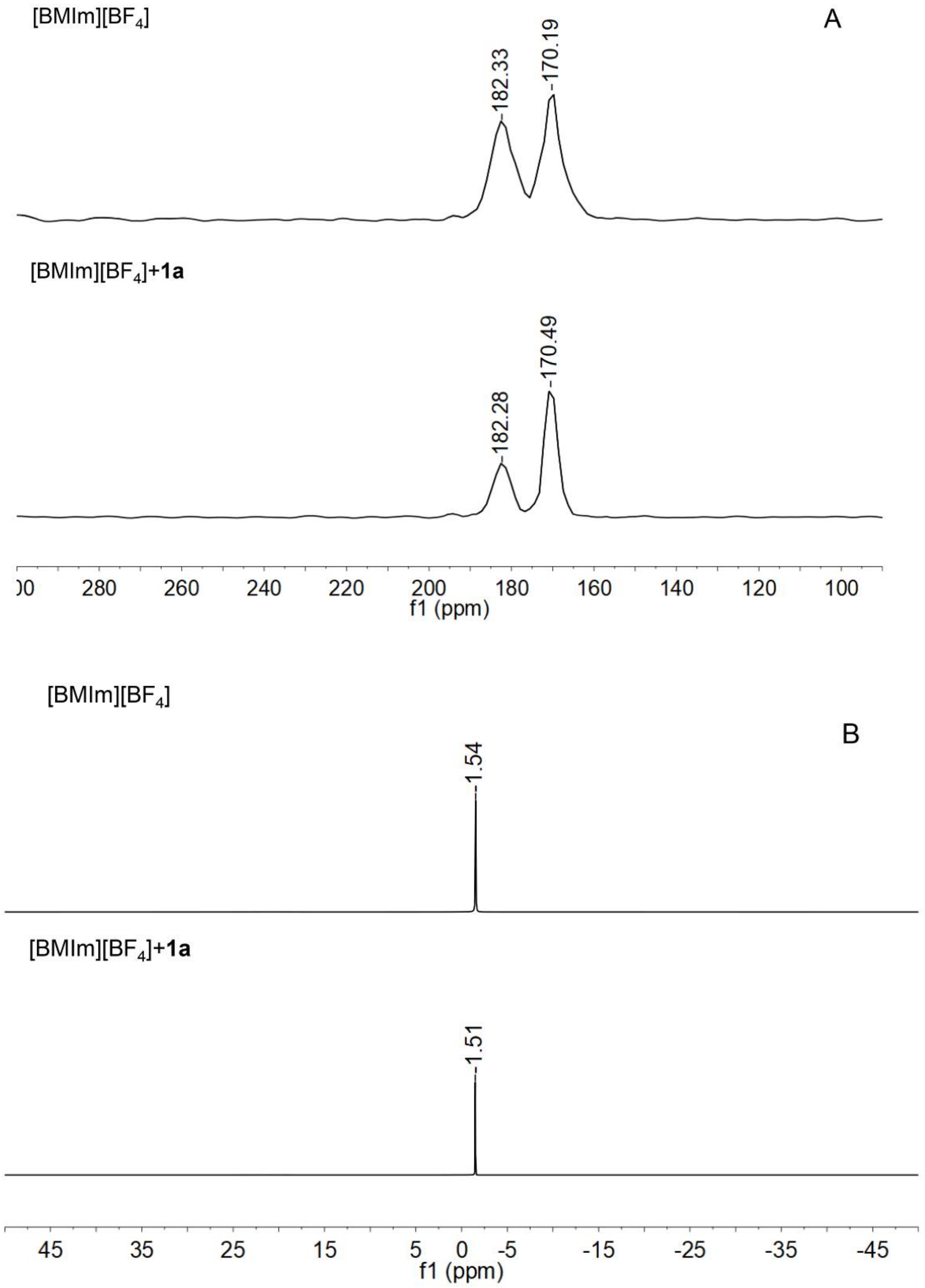

Figure S7. ^{15}N (a) and ^{11}B (b) NMR spectra of [BMIm][BF_4] and the mixture of **1a** and [BMIm][BF_4].

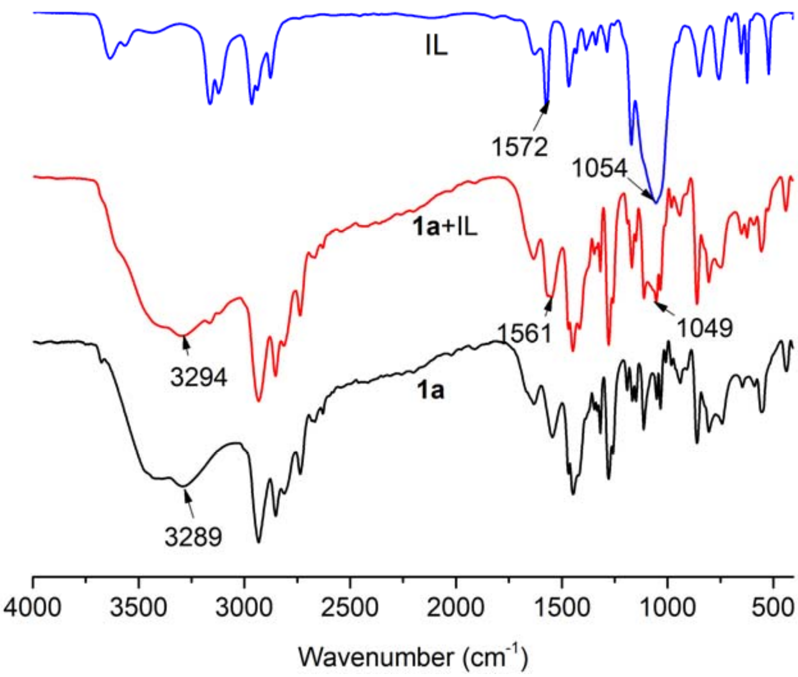

Figure S8. FTIR spectra of piperidine (**1a**), [BMIm][BF_4] (IL) and their mixture (**1a**+IL).

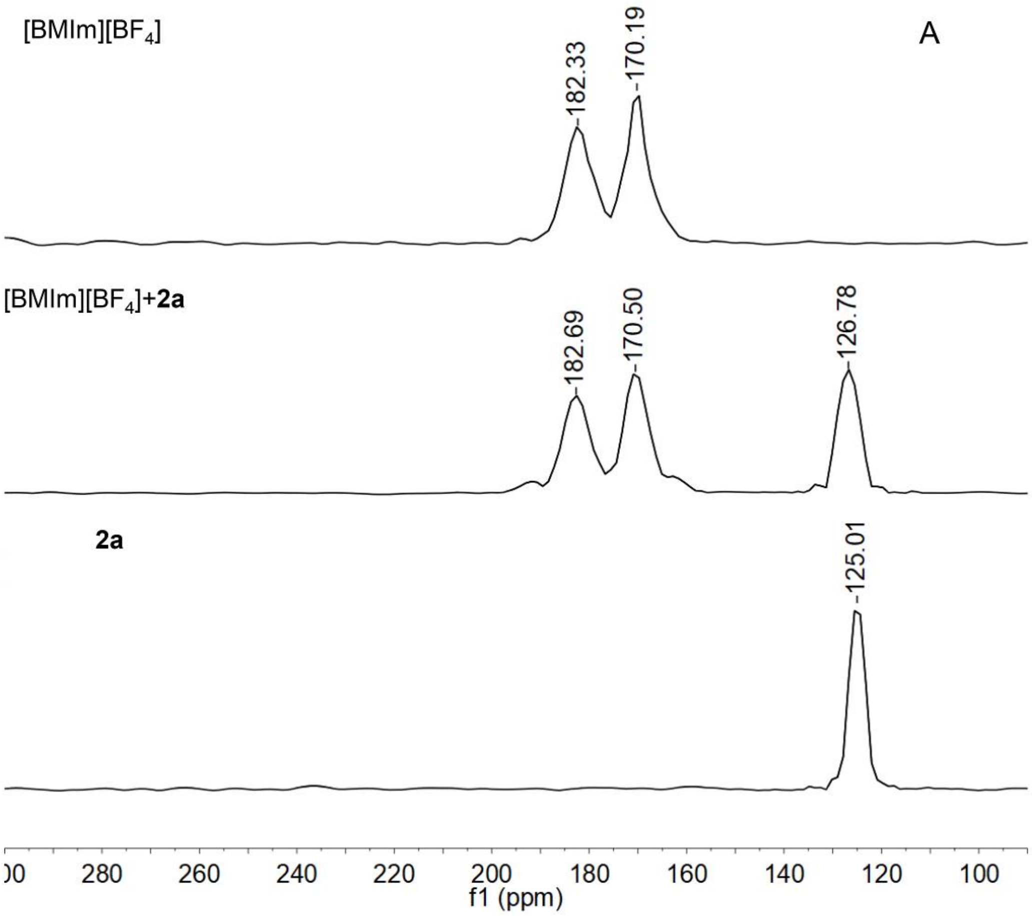

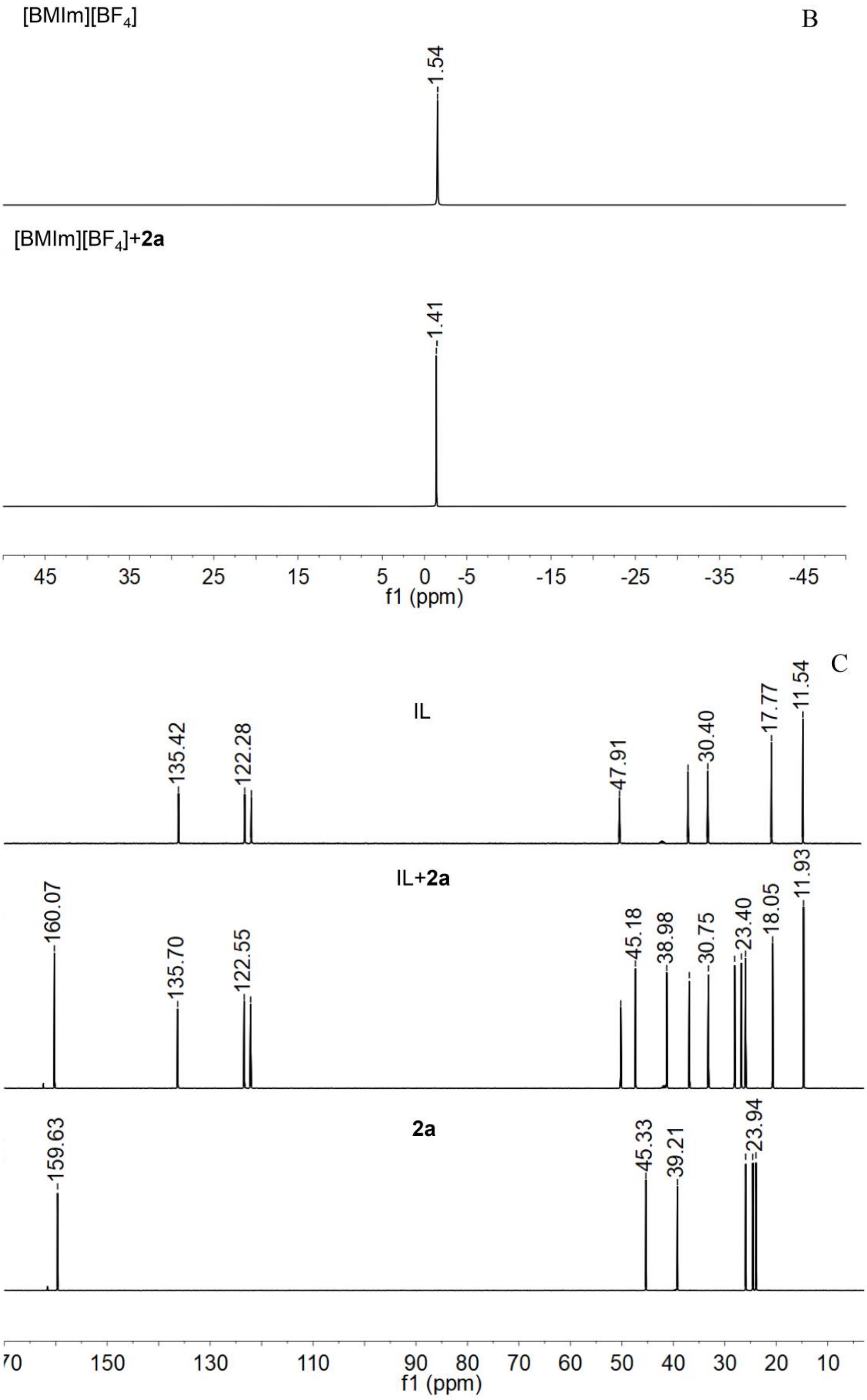

Figure S9. ^{15}N (A), ^{11}B (B) and ^{13}C NMR (C) spectra of [BMIm][BF_4] and the mixture of **2a** and [BMIm][BF_4].

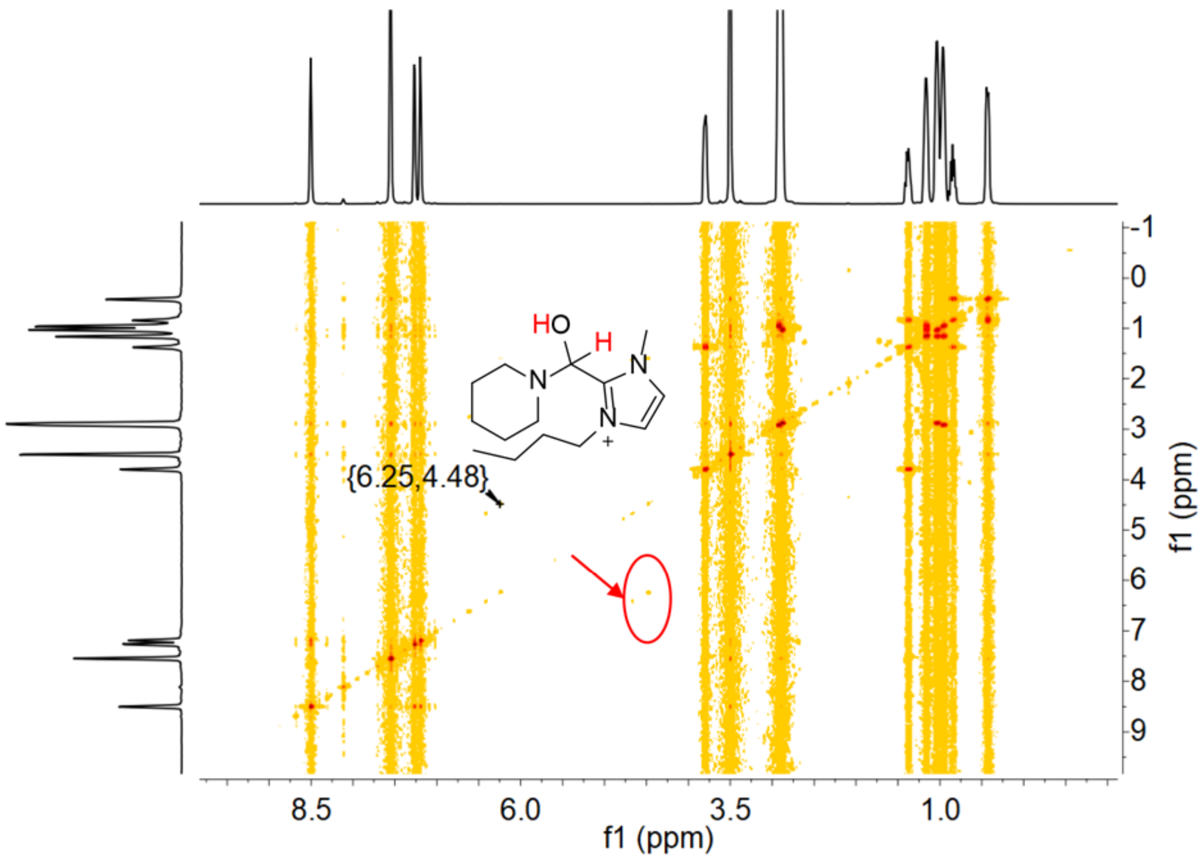

Figure S10. ^1H-^1H COSY spectrum of the mixture of IL and **2a** together with K_2CO_3.

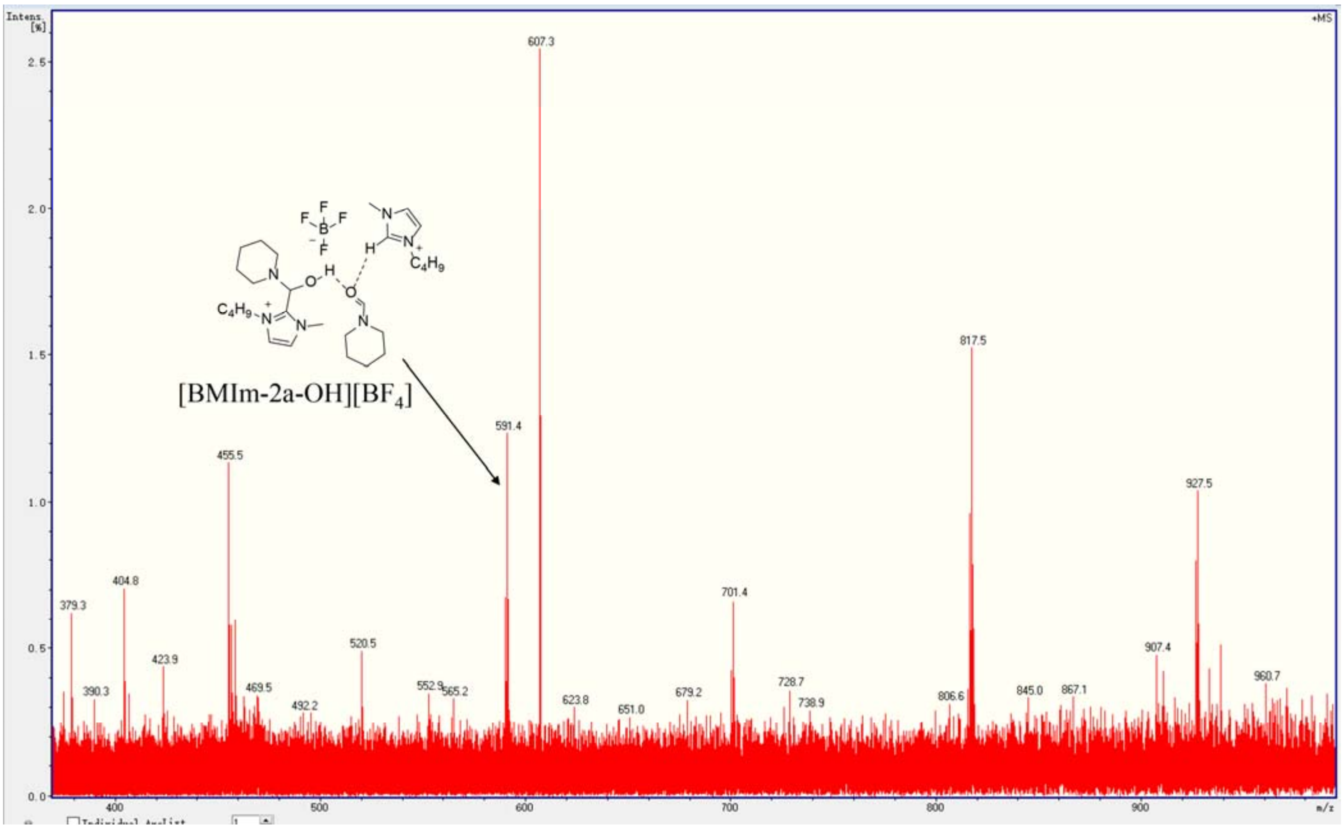

Figure S11. Electrospray ionization mass spectrometry(ESI-MS) of the reaction mixture of **2a** with IL with K_2CO_3 after 1 h in positive ion mode.

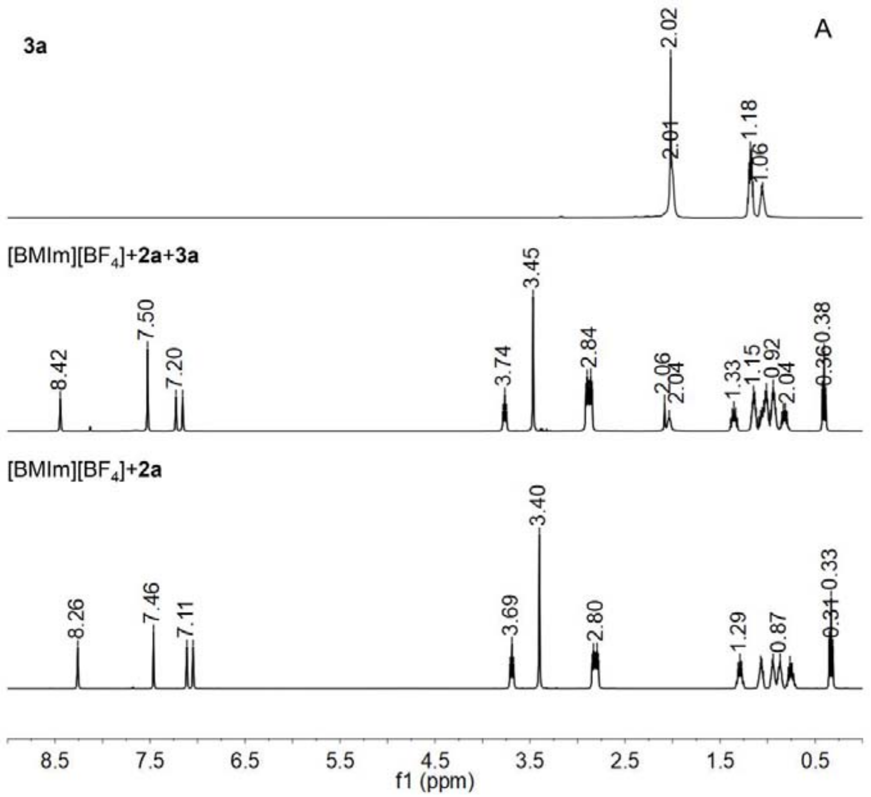

Figure S12. ^{1}H NMR (A) and ^{13}C NMR (B) spectra of 1,2-bis(piperidine)ethane (**3a**), the mixture of [BMIm][BF_4], **2a** and **3a**, and the mixture of **2a** and [BMIm][BF_4].

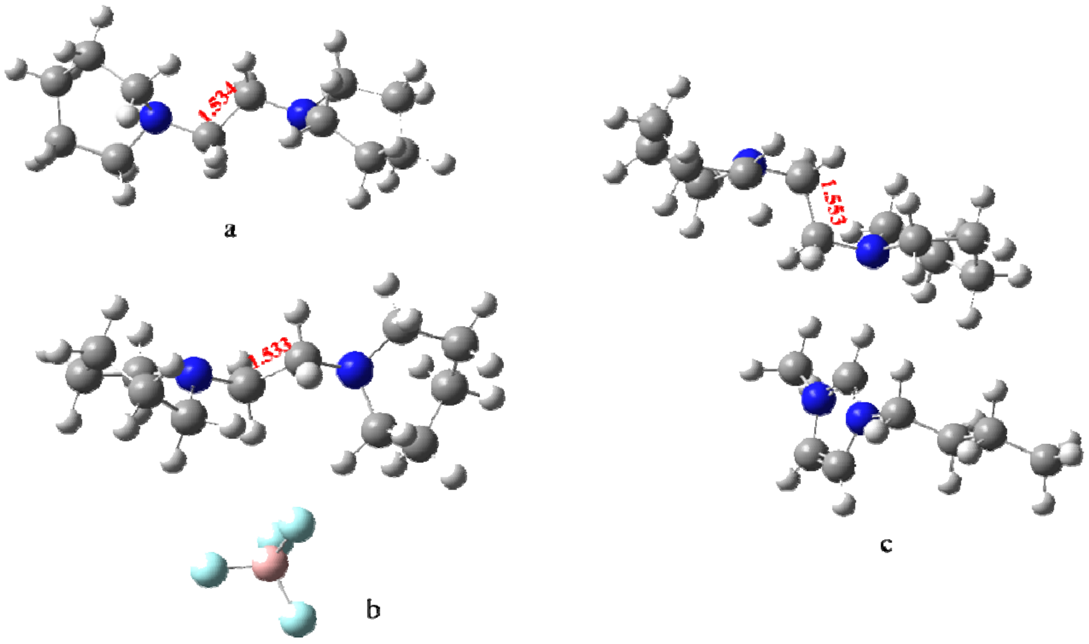

Figure S13. The structures of **3a** (a), **3a** with [BF_4] (b) and with [BMIm] (c). Red word: atom distance (Å).

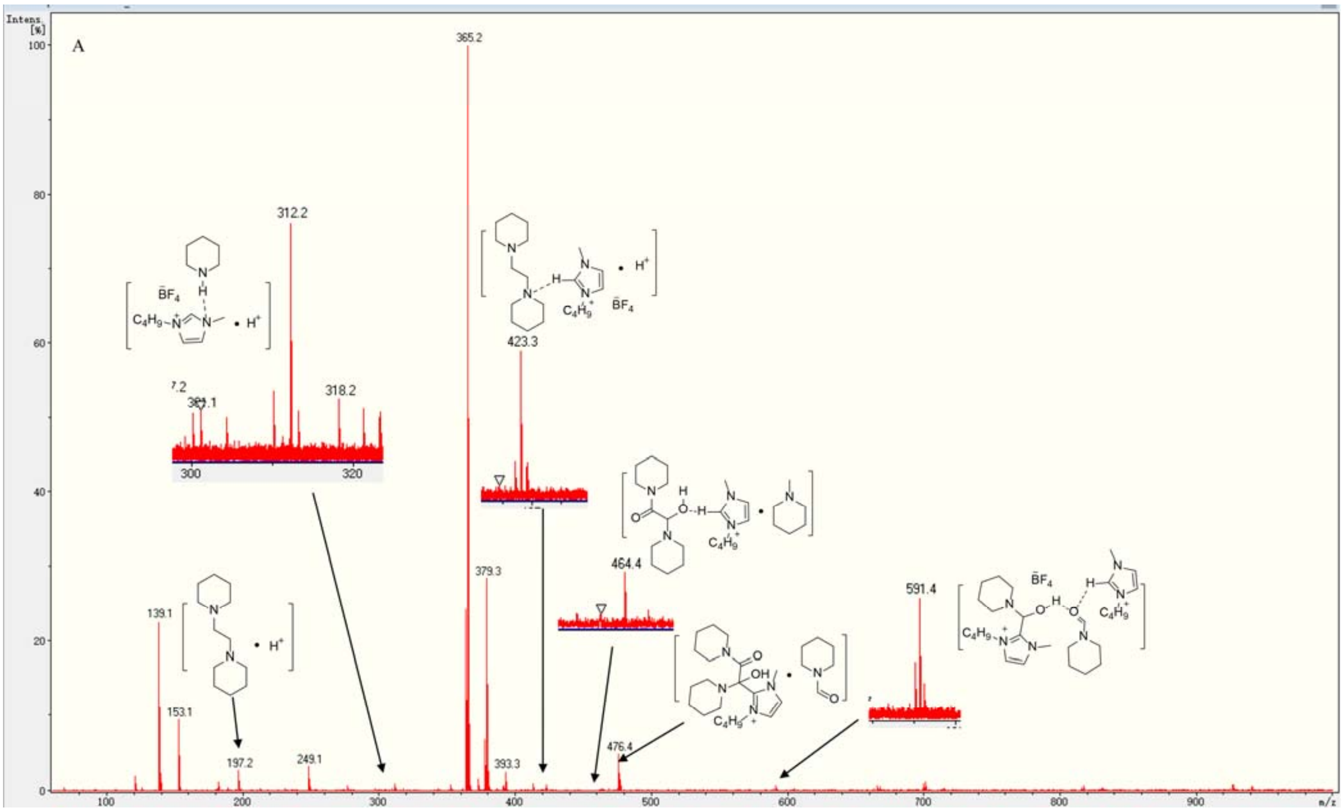

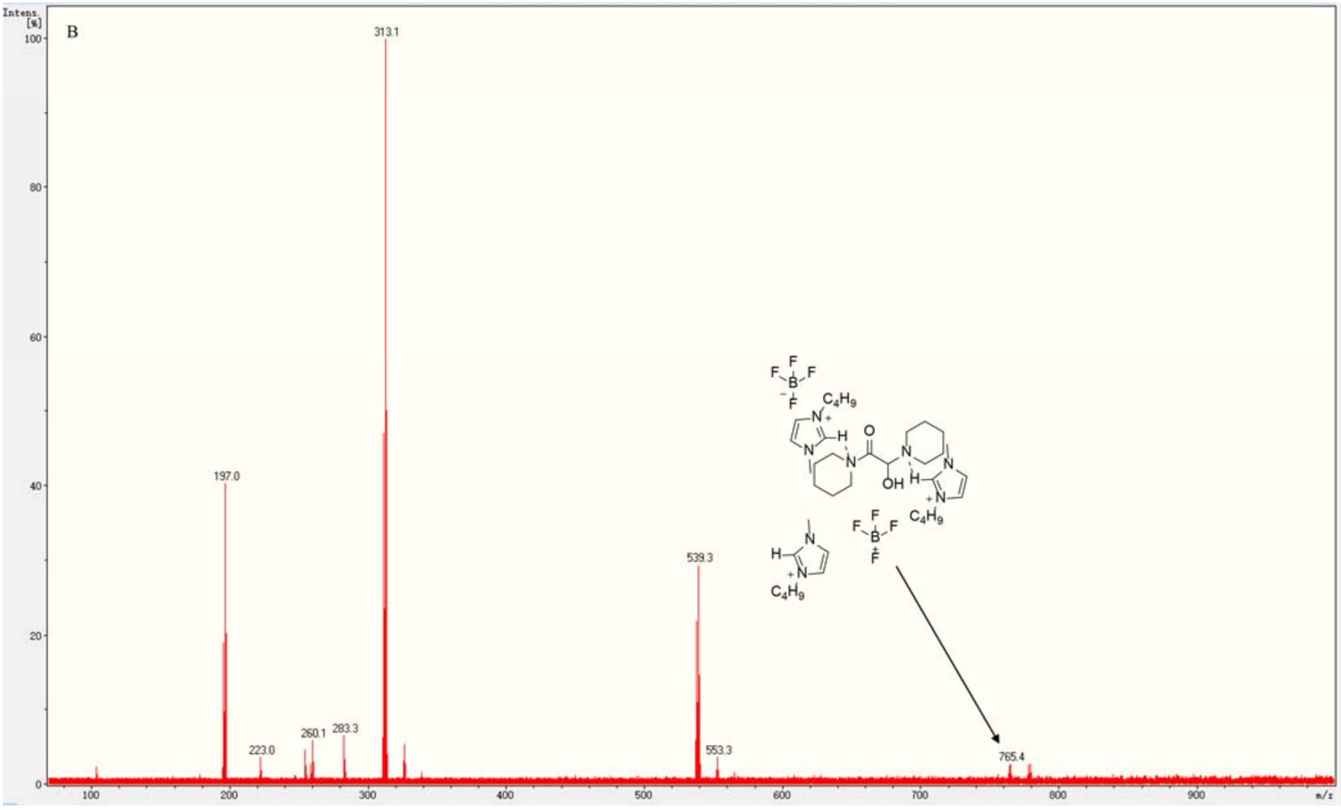

Figure S14. ESI-MS analysis of the reaction mixture after 1 h in positive ion mode (A) and negative ion mode (B).

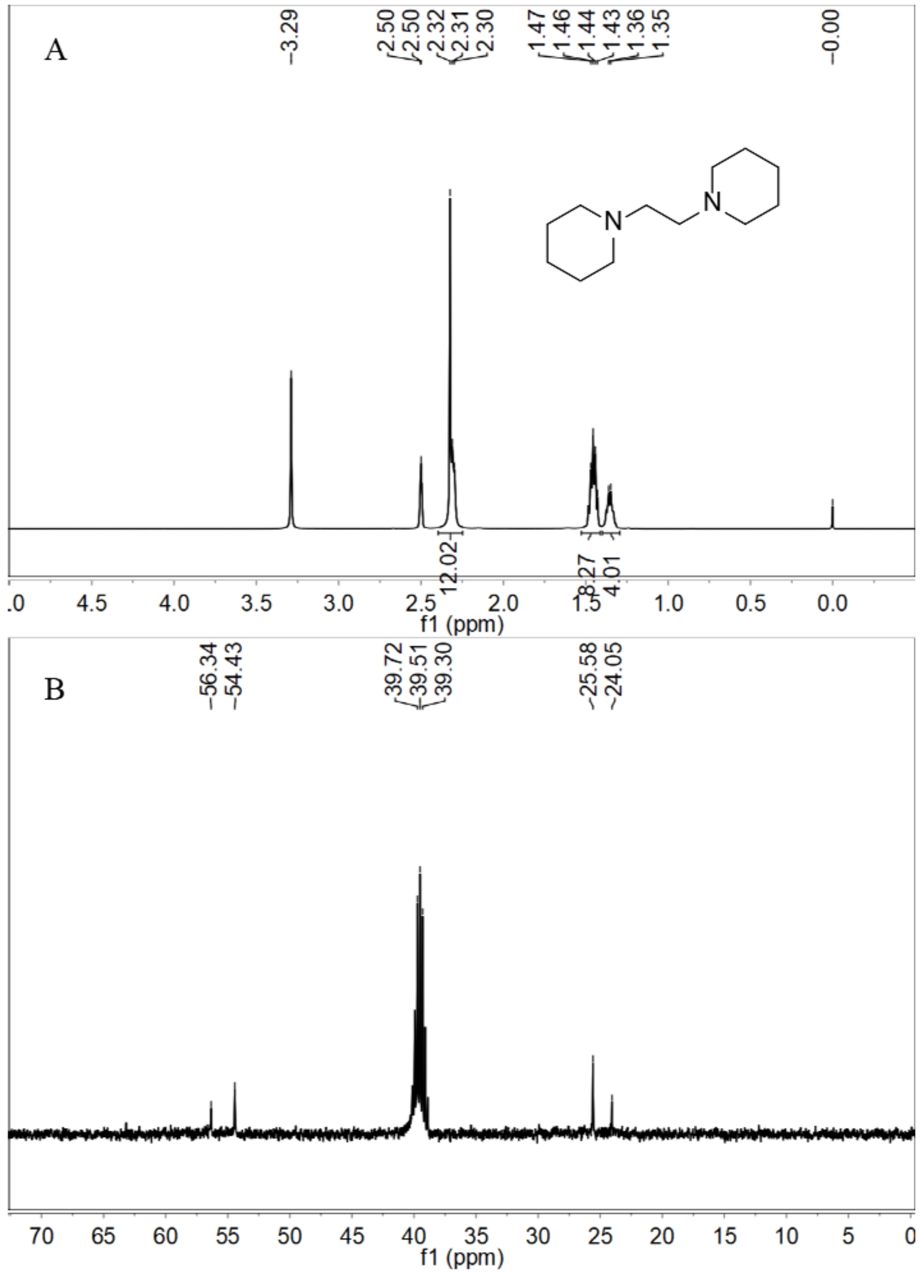

Figure S15. ^{1}H NMR (A) and ^{13}C NMR (B) spectra of 1,2-bis(piperidine)ethane.

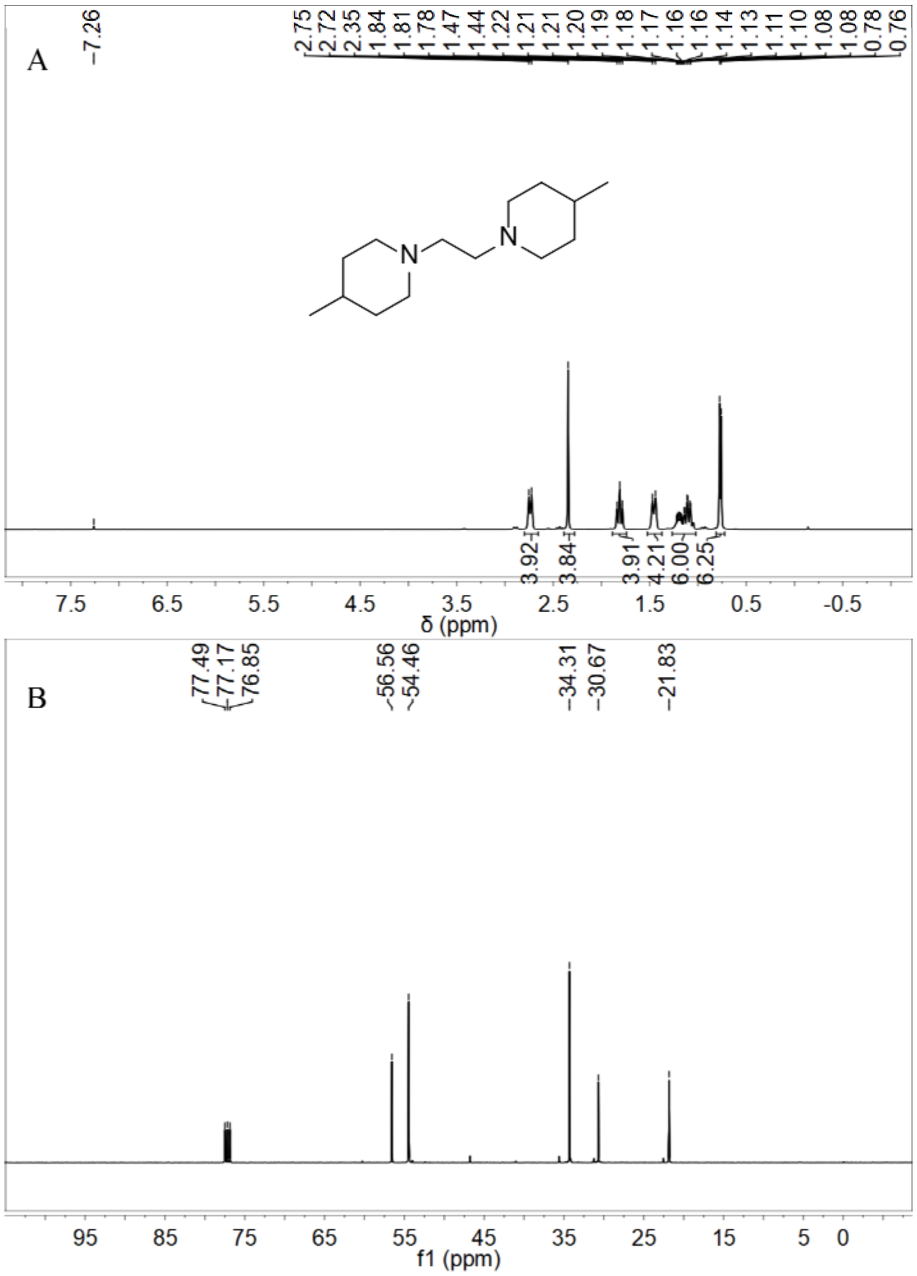

Figure S16. ^{1}H NMR (A) and ^{13}C NMR (B) spectra of 1,2-bis(4-methylpiperidin-1-yl)ethane.

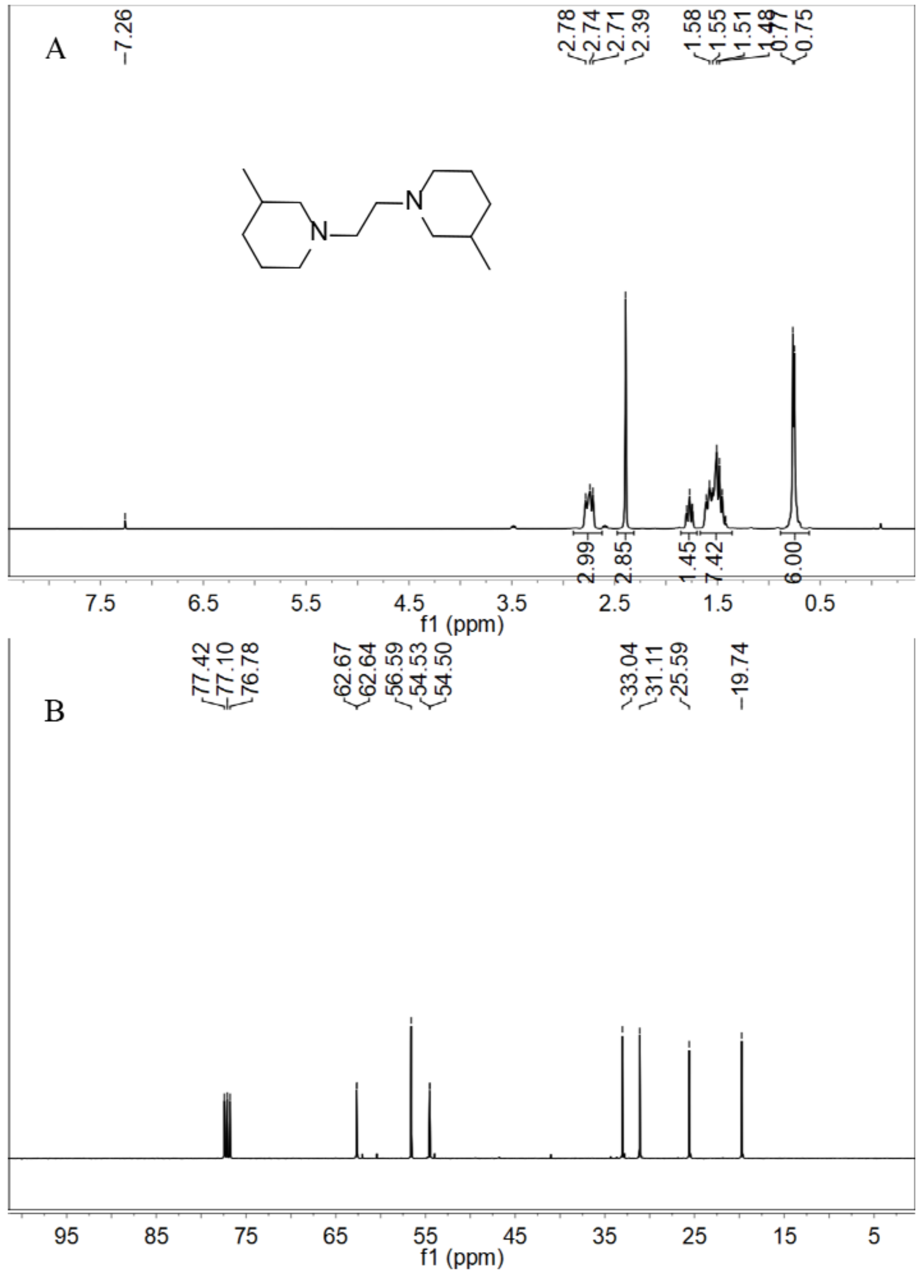

Figure S17. ^{1}H NMR (A) and ^{13}C NMR (B) spectra of 1,2-bis(3-methylpiperidin-1-yl)ethane.

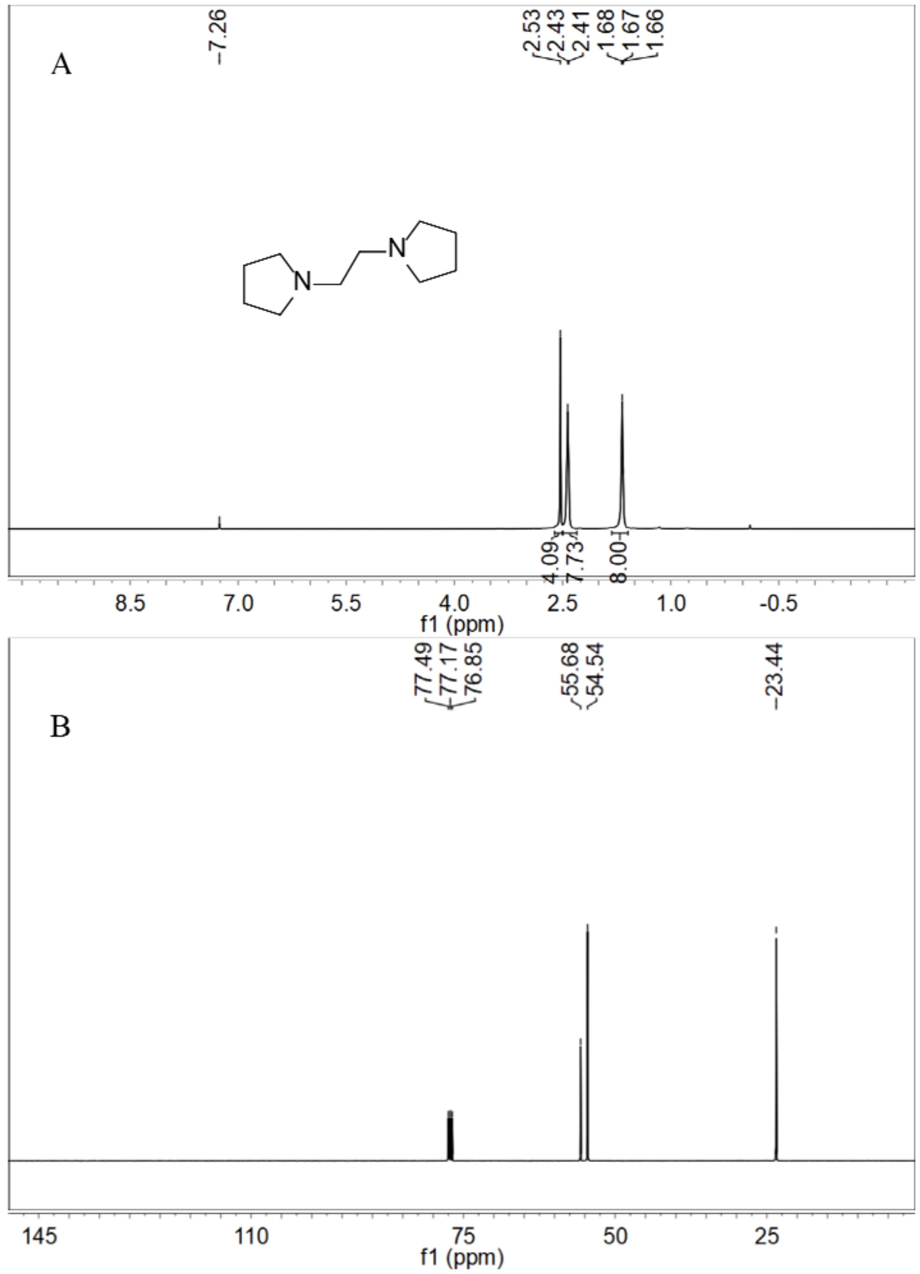

Figure S18. ^{1}H NMR (A) and ^{13}C NMR (B) spectra of 1,2-di(pyrrolidin-1-yl)ethane.

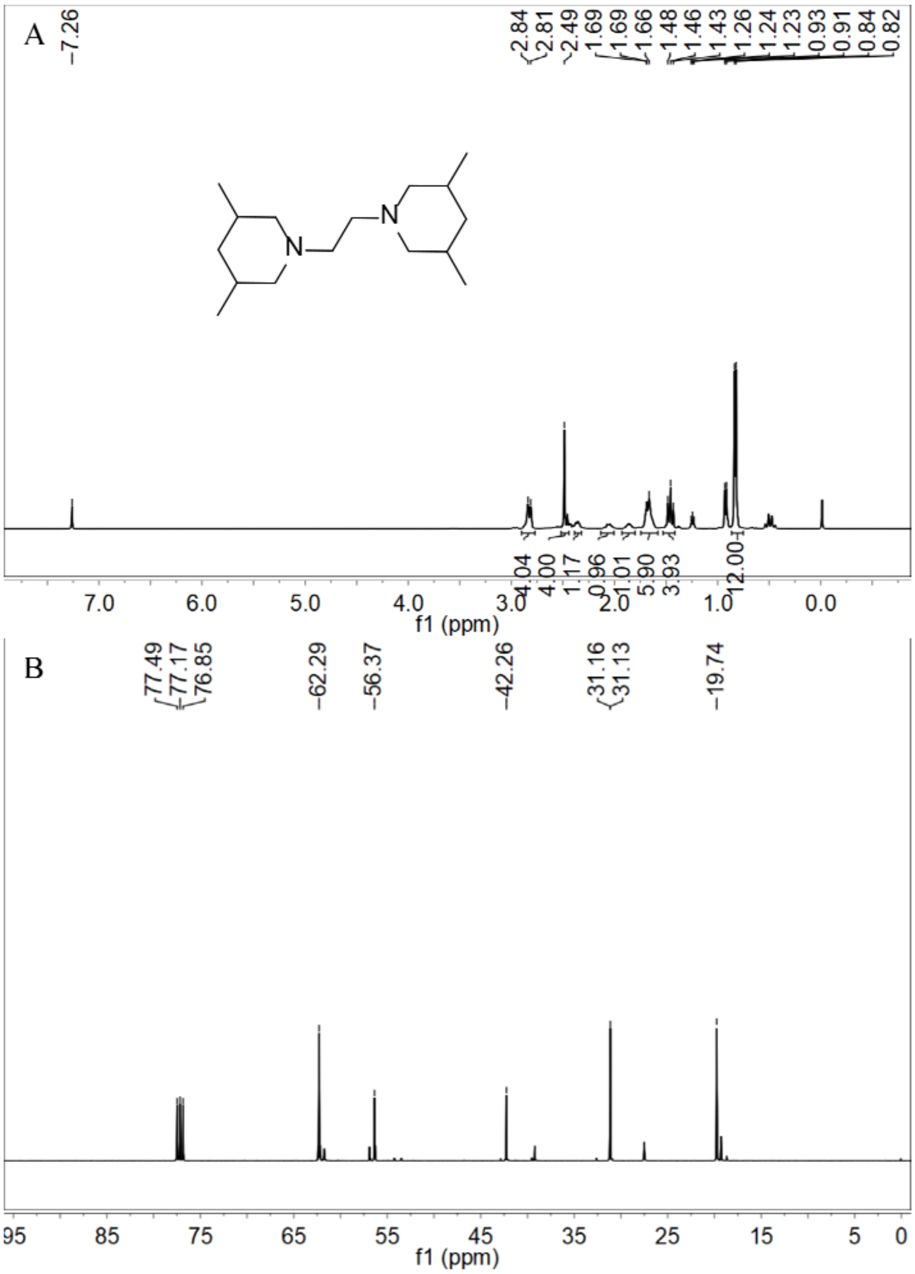

Figure S19. ^{1}H NMR (A) and ^{13}C NMR (B) spectra of 1,2-bis(3,5-dimethylpiperidin-1-yl)ethane.

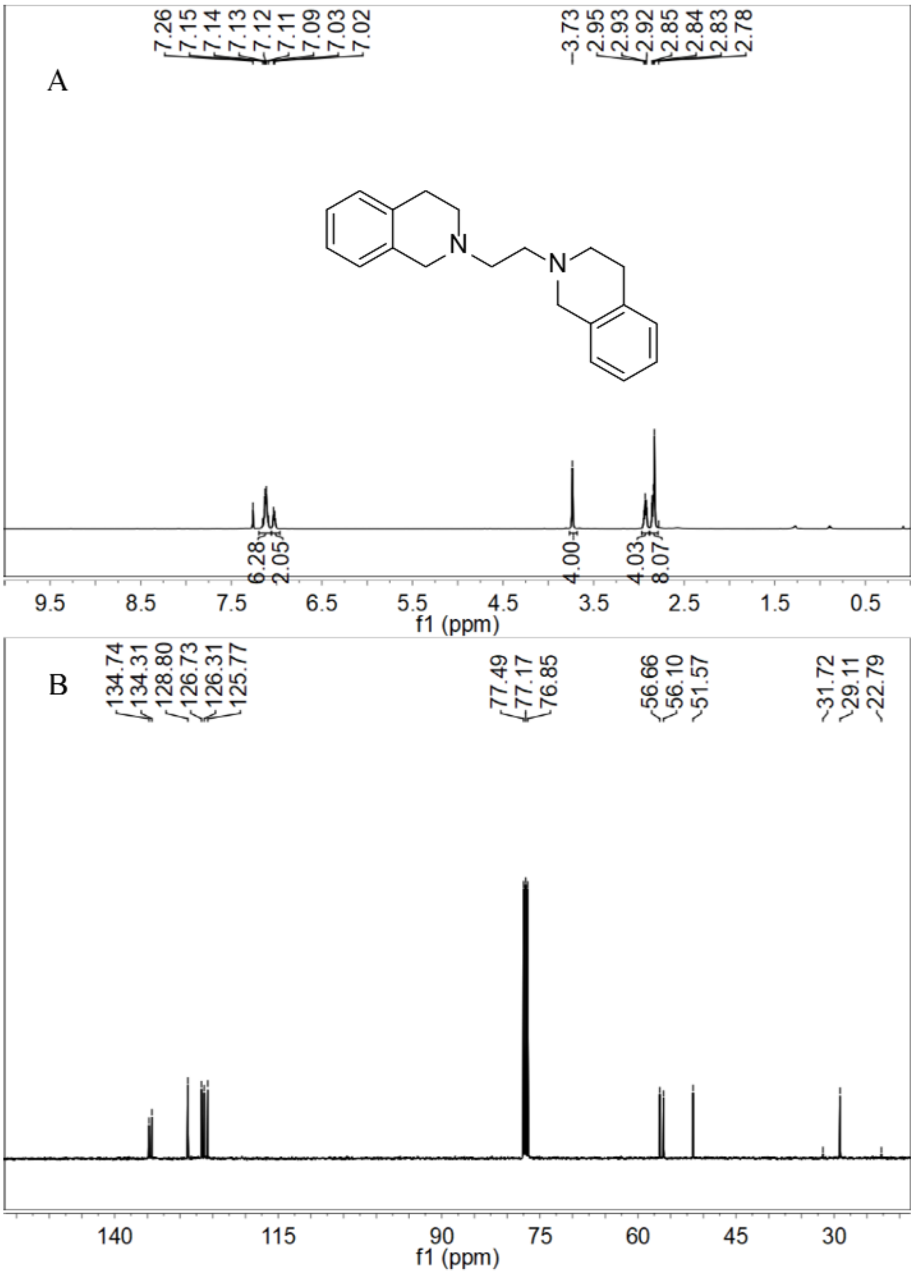

Figure S20. ^{1}H NMR (A) and ^{13}C NMR (B) spectra of 1,2-bis-(3,4-dihydro-1H-[2]isoquinolyl)-ethane.

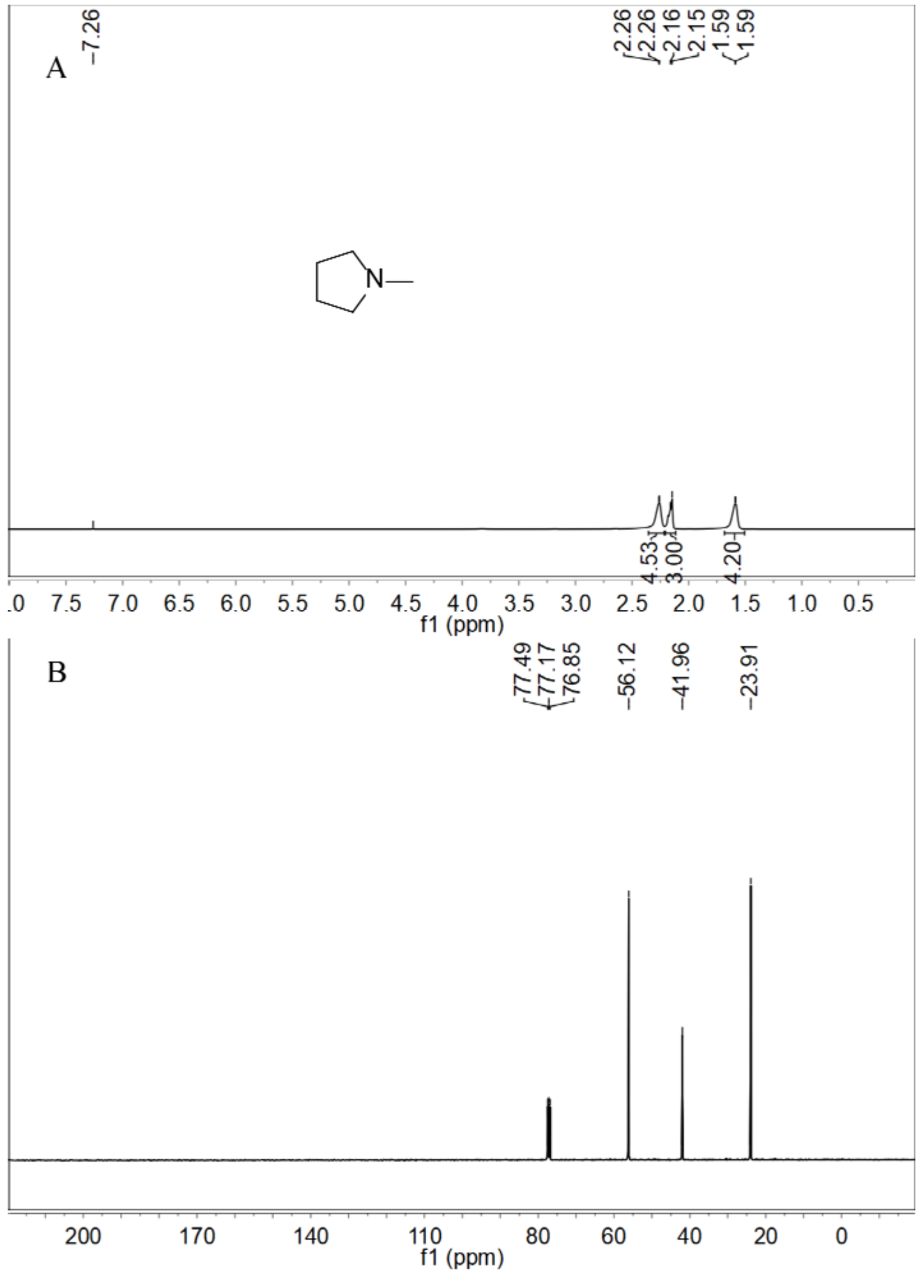

Figure S21. ^{1}H NMR (A) and ^{13}C NMR (B) spectra of 1-methylpyrrolidine.

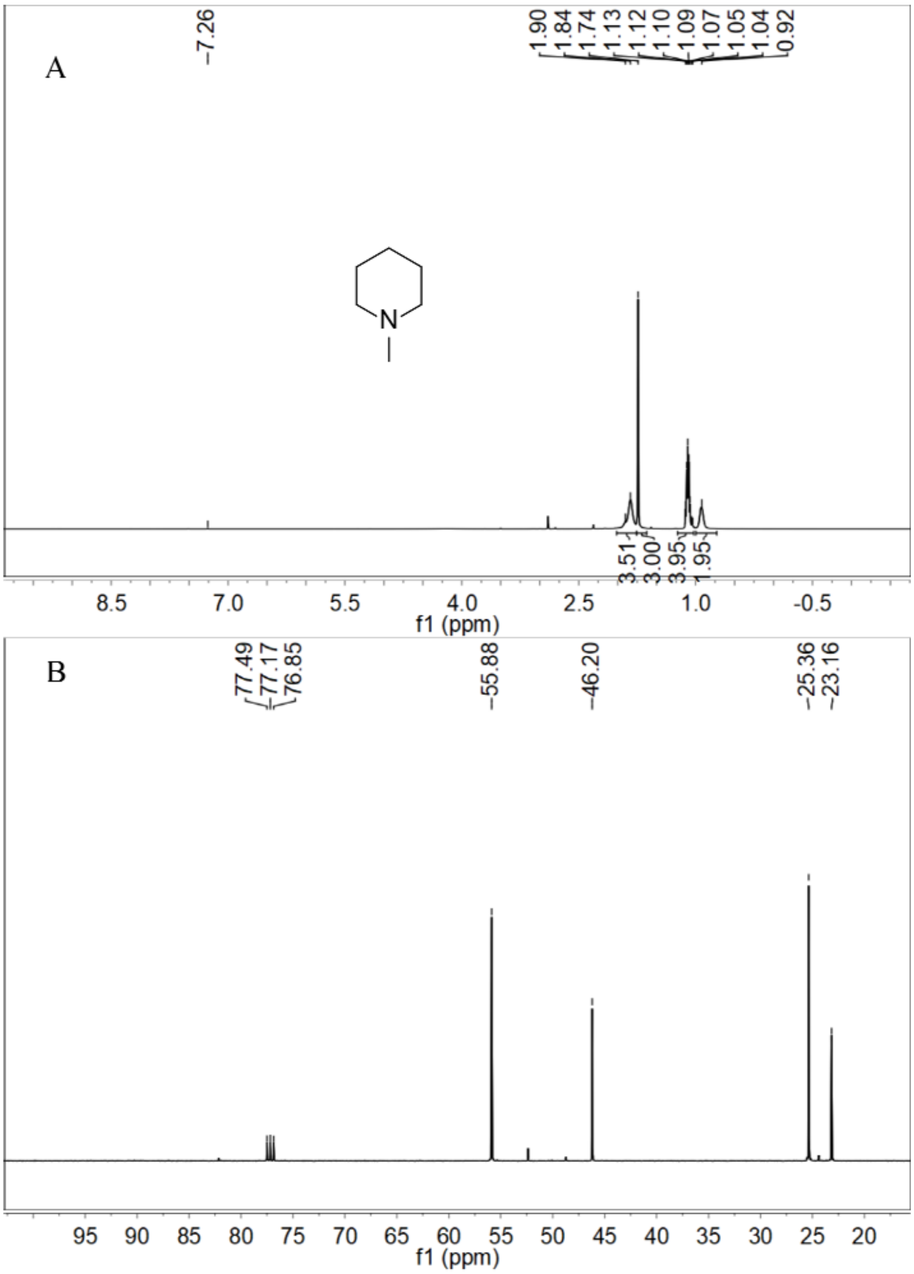

Figure S22. ^{1}H NMR (A) and ^{13}C NMR (B) spectra of N-methylpiperidine.

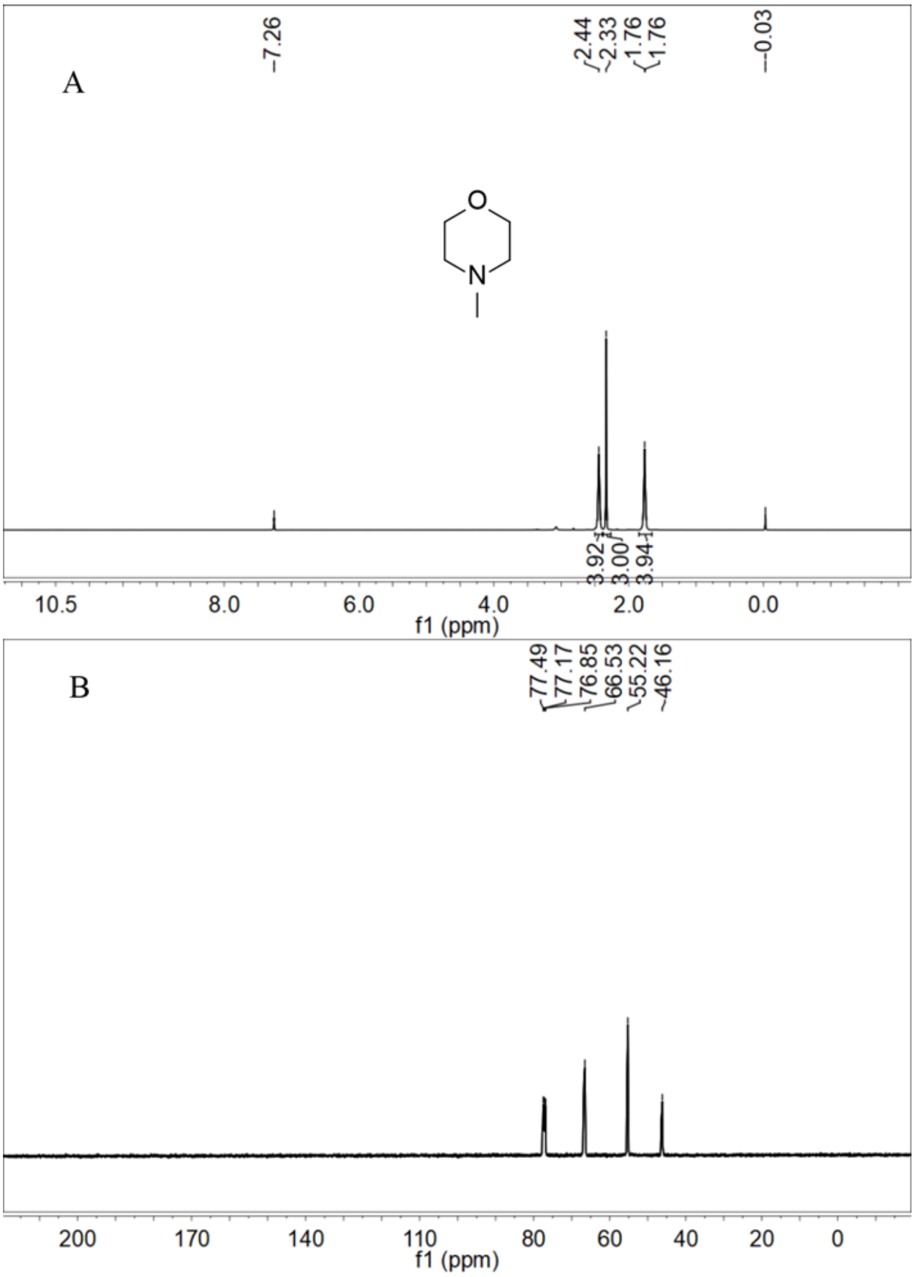

Figure S23. ^{1}H NMR (A) and ^{13}C NMR (B) spectra of 4-methyl-morpholine.

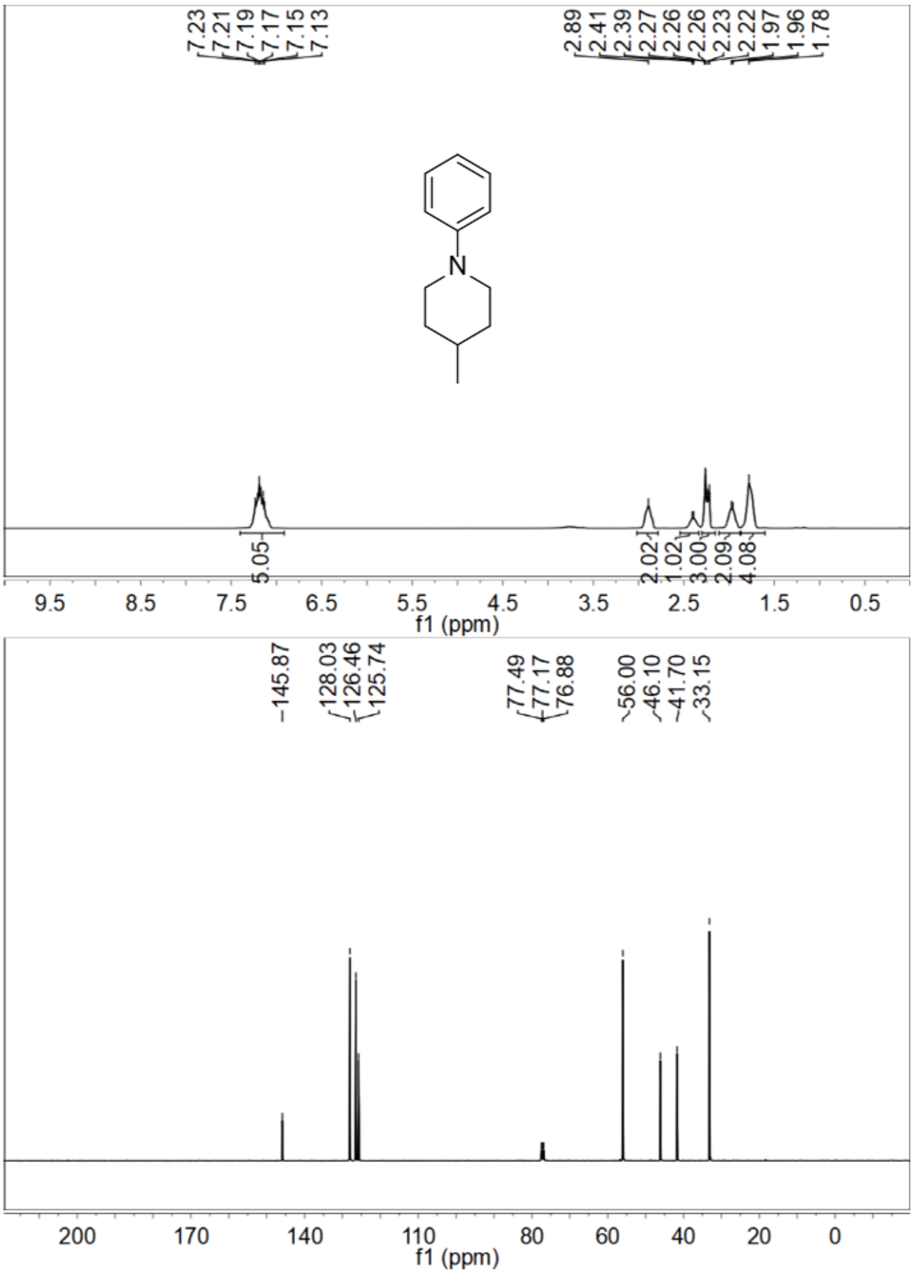

Figure S24. ^{1}H NMR (A) and ^{13}C NMR (B) spectra of 1-methyl-4-phenylpiperidine.

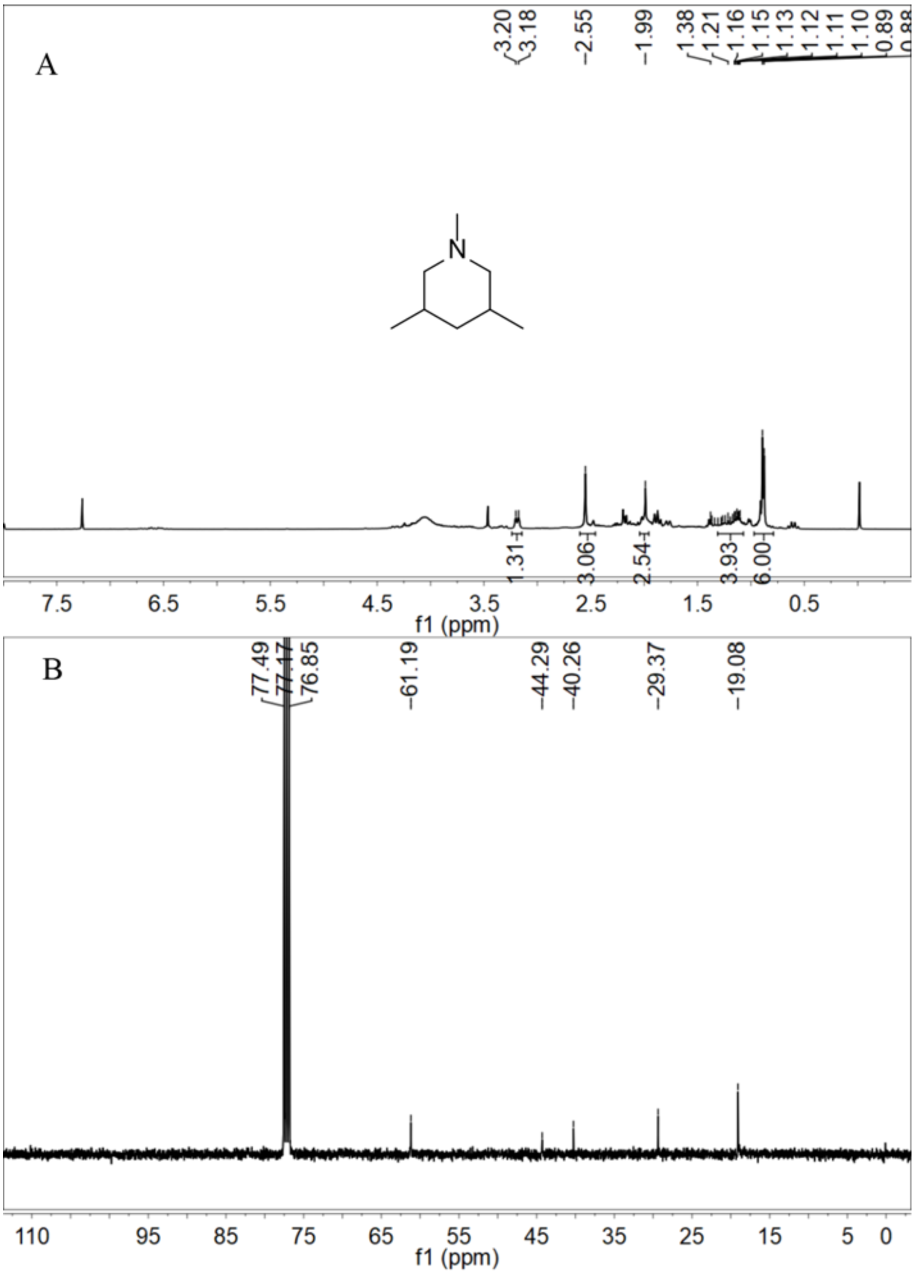

Figure S25. ^{1}H NMR (A) and ^{13}C NMR (B) spectra of 1,3,5-trimethylpiperidine.

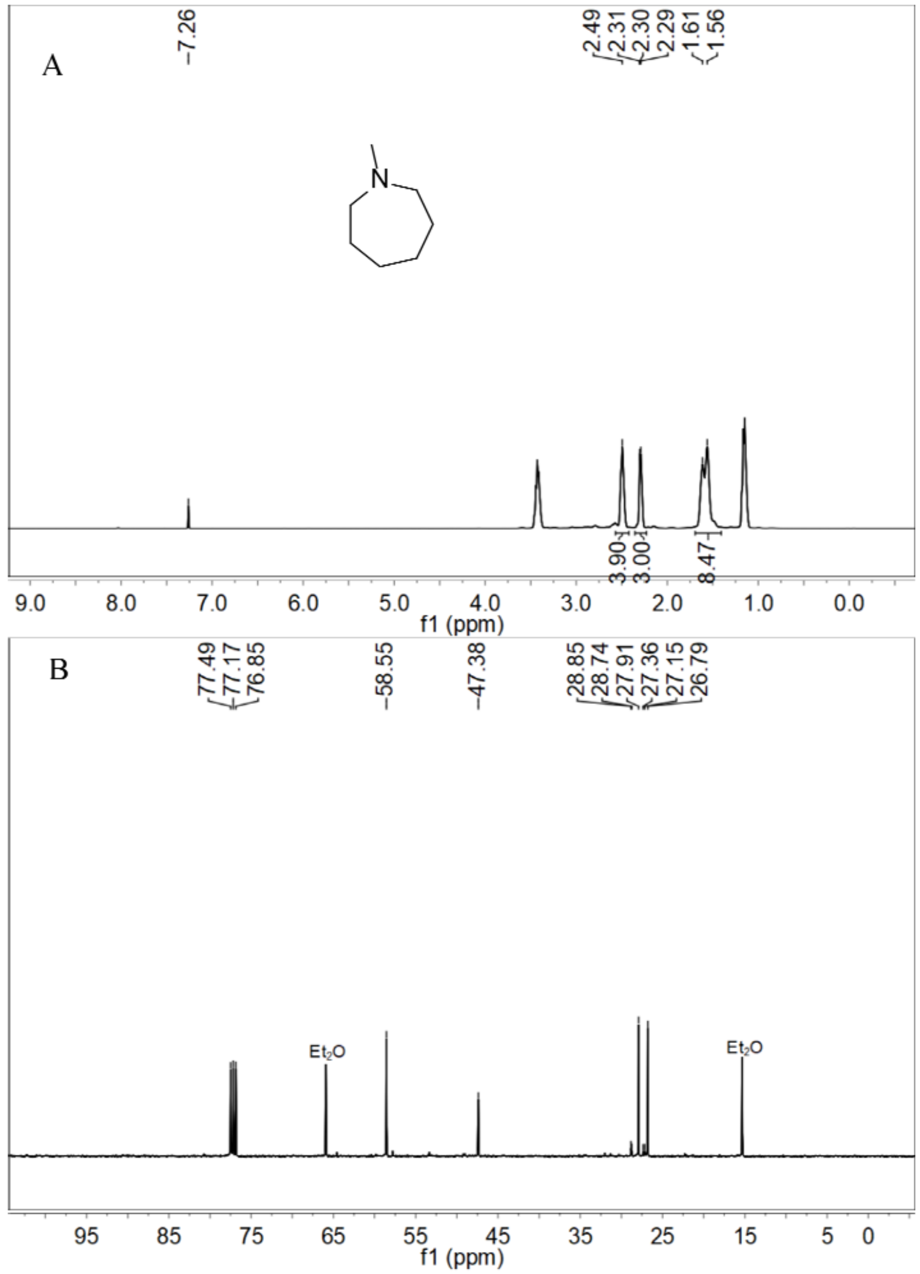

Figure S26. ^{1}H NMR (A) and ^{13}C NMR (B) spectra of hexahydro-1-methyl-1H-azepine.

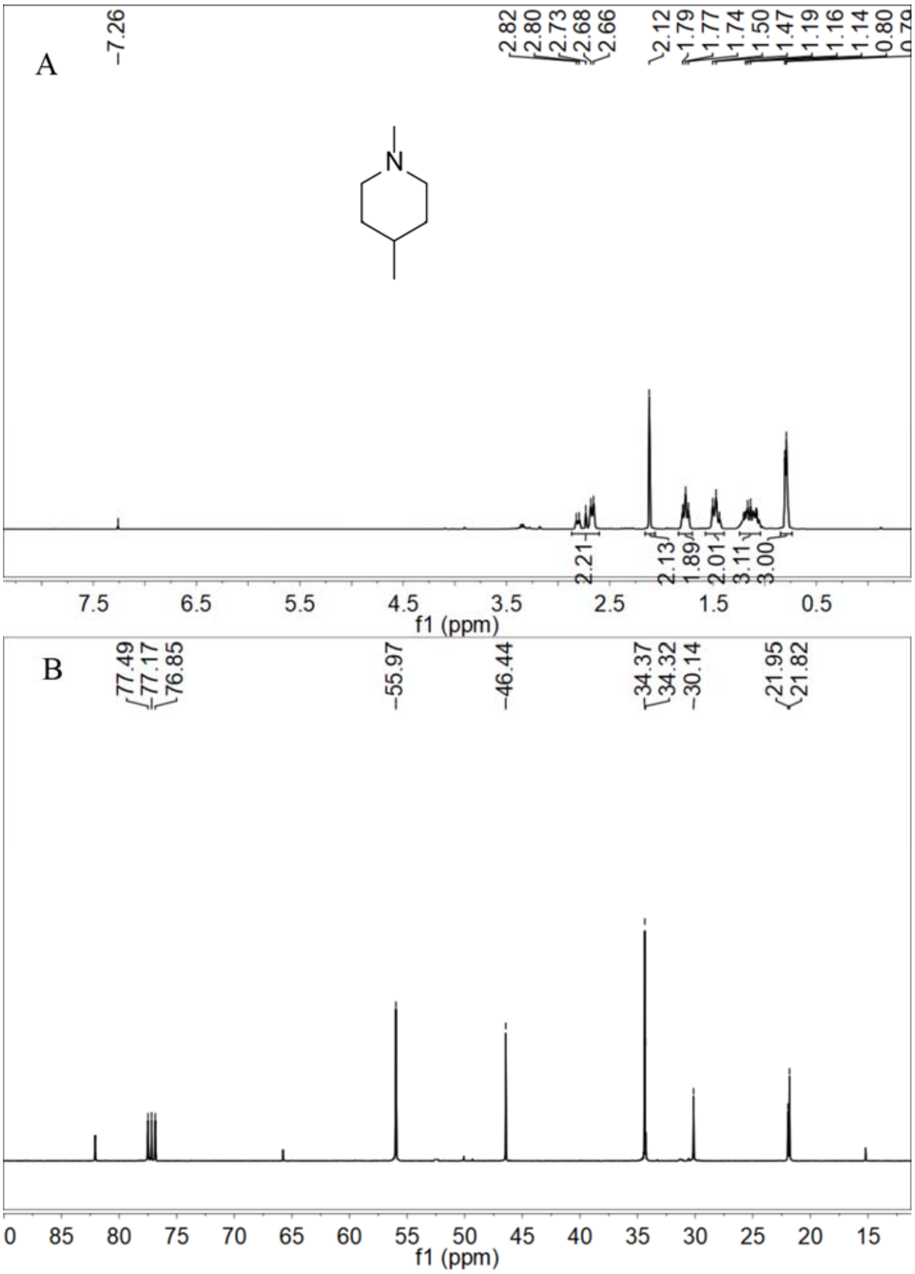

Figure S27. ^{1}H NMR (A) and ^{13}C NMR (B) spectra of 1,4-dimethylpiperidine.

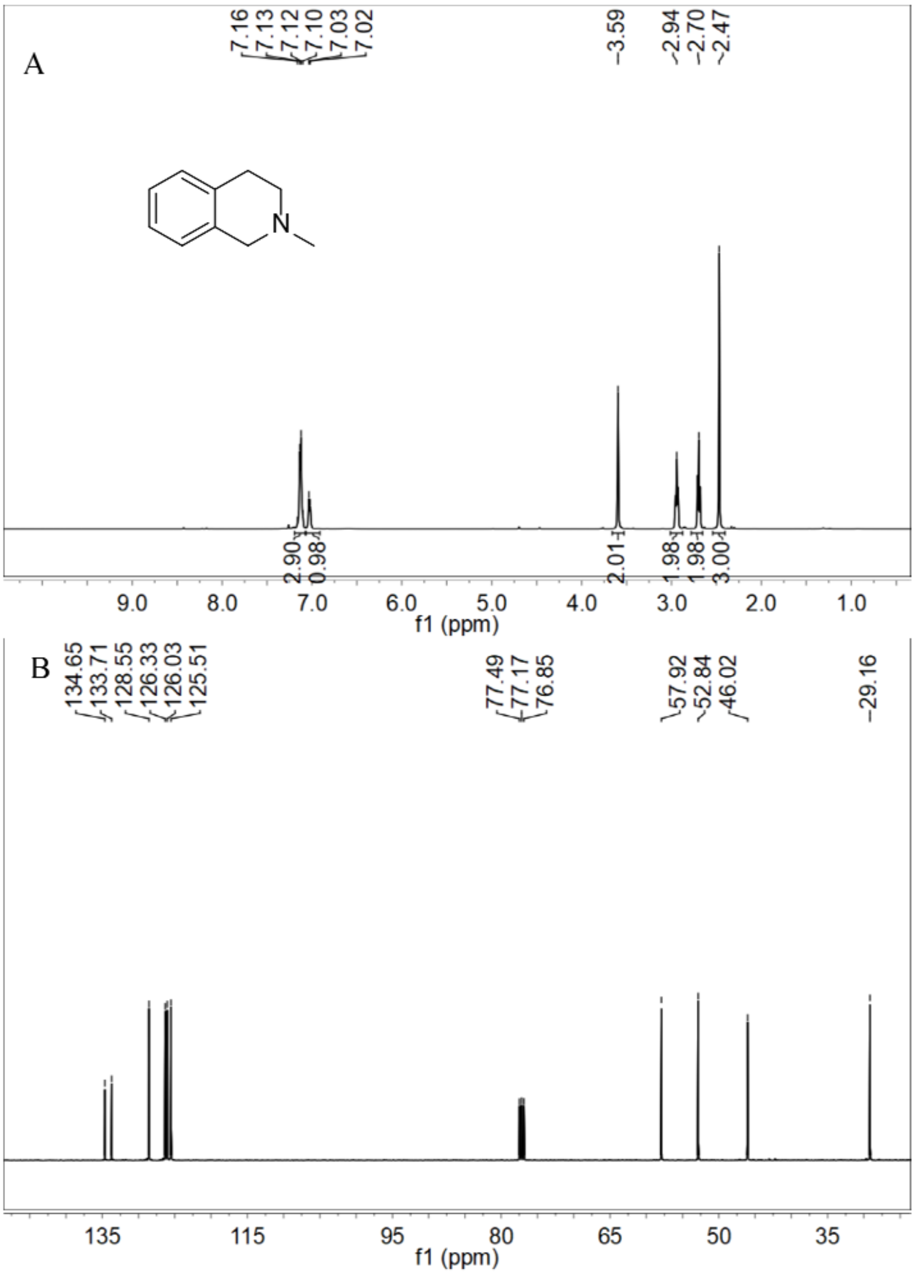

Figure S28. ^{1}H NMR (A) and ^{13}C NMR (B) spectra of 2-methyl-1,2,3,4-tetrahydroisoquinoline

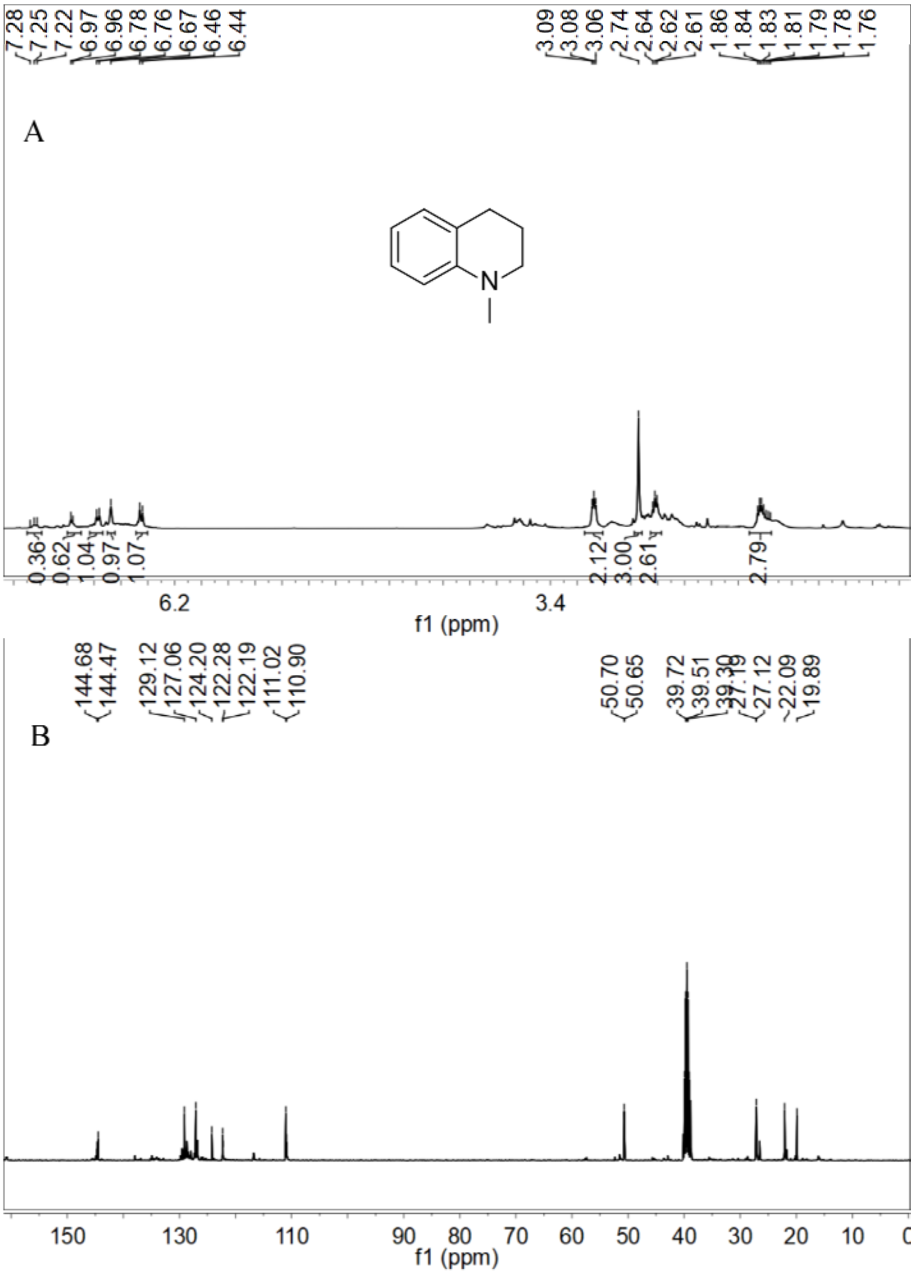

Figure S29. ^{1}H NMR (A) and ^{13}C NMR (B) spectra of N-methyl-1,2,3,4-tetrahydroquinoline.

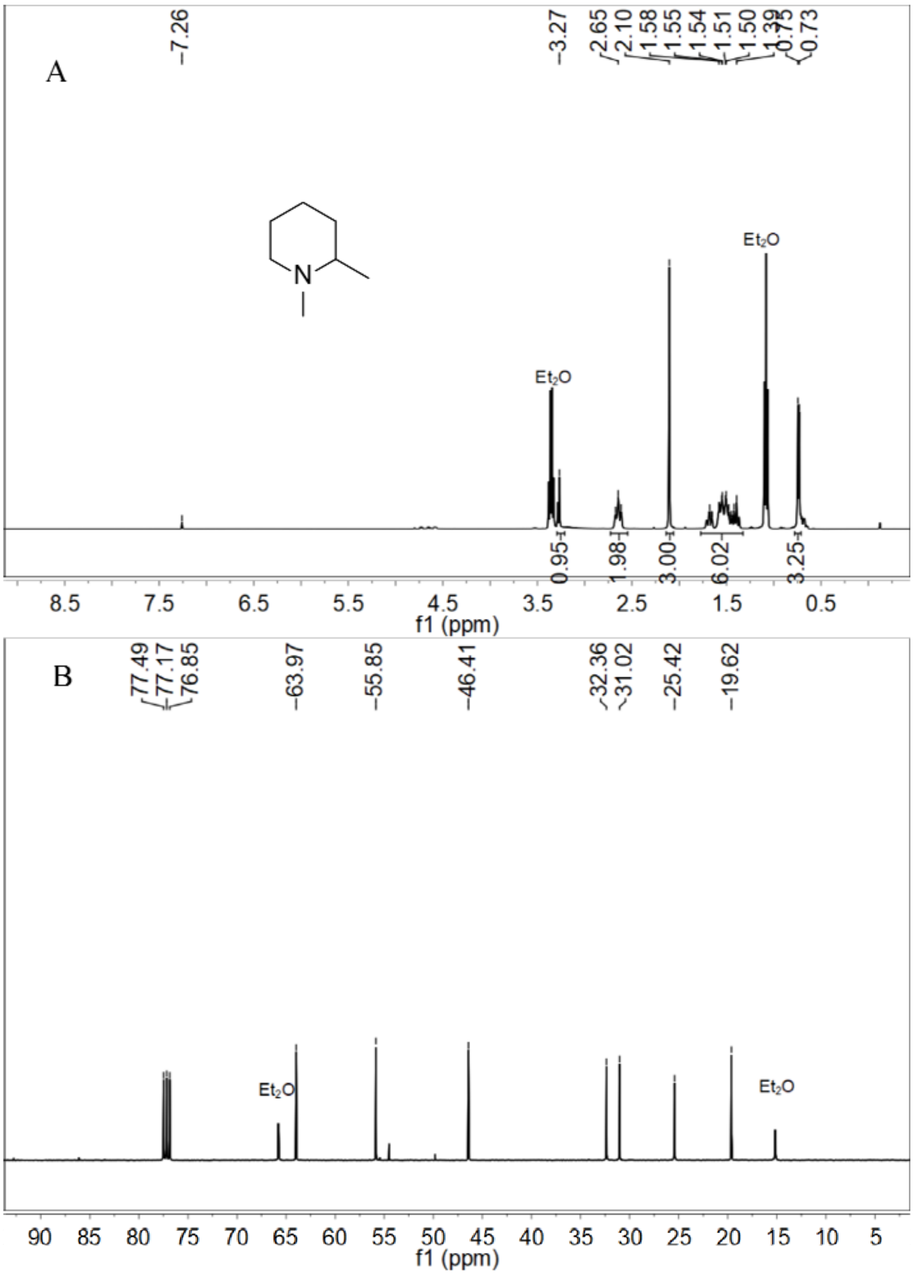

Figure S30. ^{1}H NMR (A) and ^{13}C NMR (B) spectra of 1,2-dimethylpiperidine.

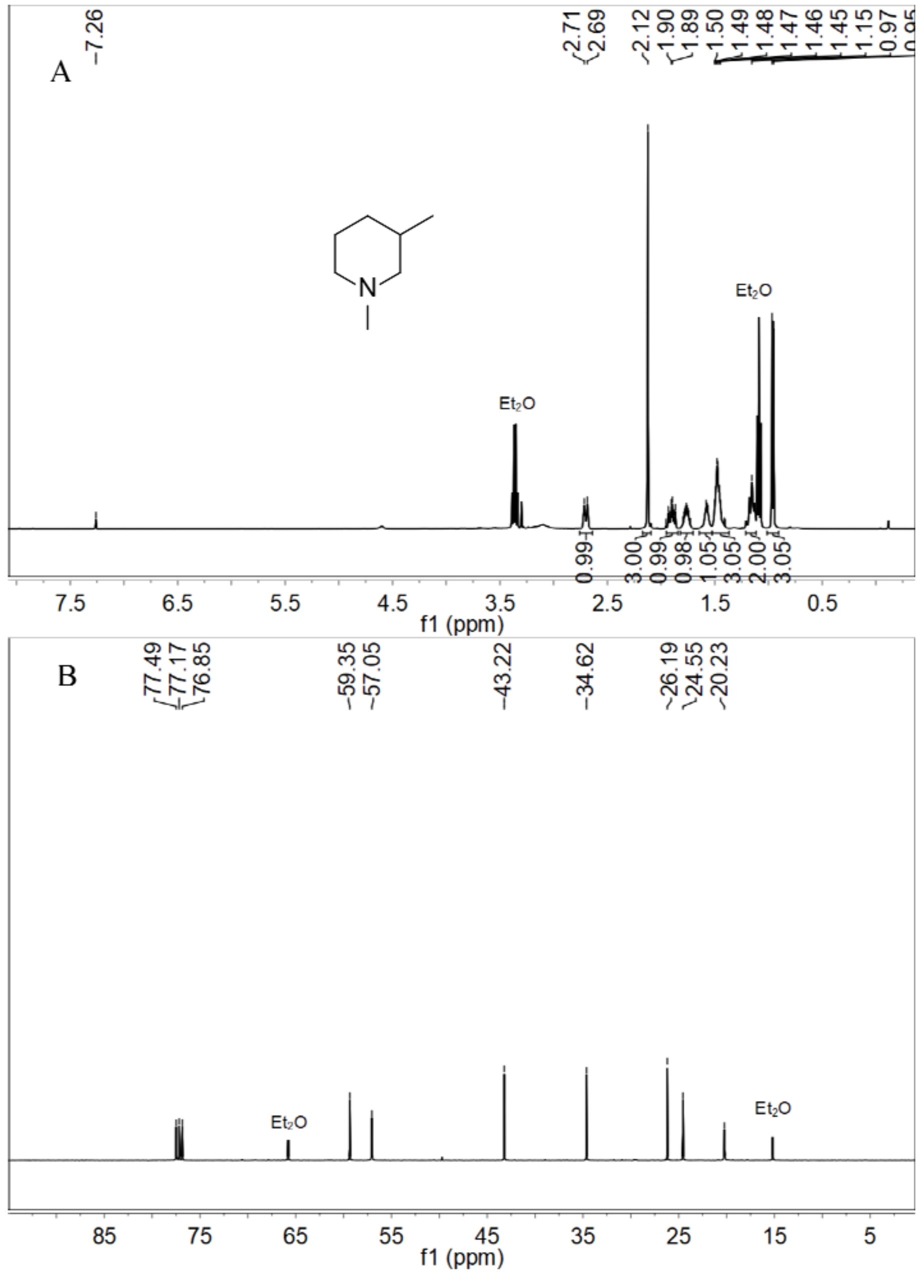

Figure S31. ^{1}H NMR (A) and ^{13}C NMR (B) spectra of 1,3-dimethylpiperidine.

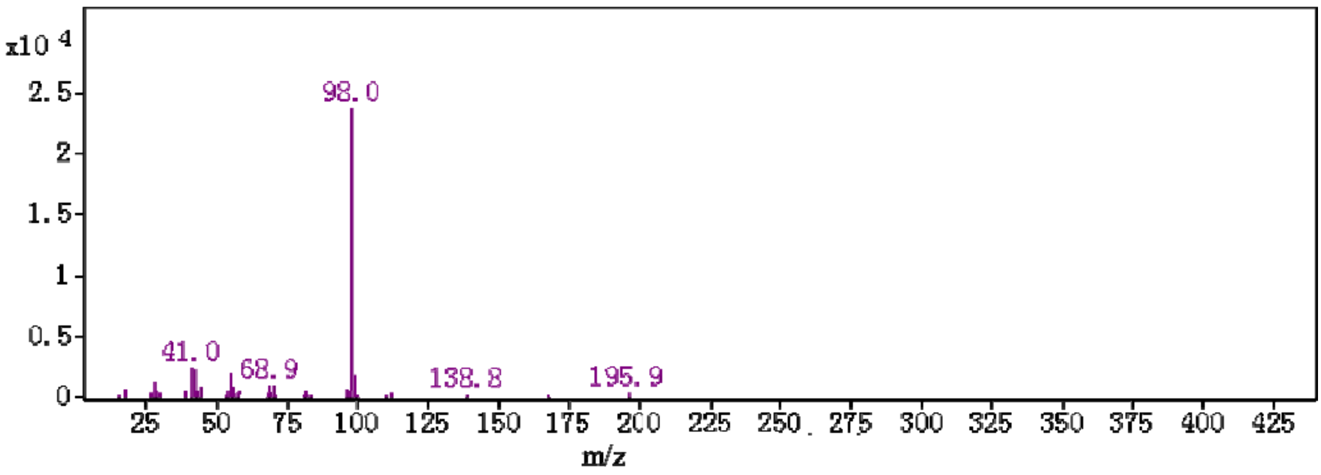

Figure S32. GC-MS spectrum of 1,2-bis(piperidine)ethane.

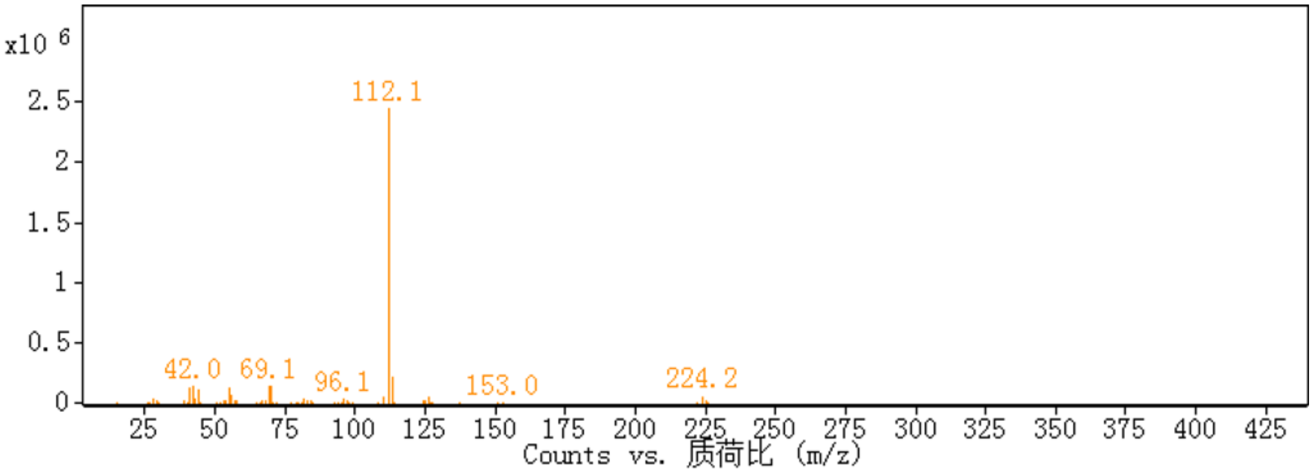

Figure S33. GC-MS spectrum of 1,2-bis(4-methylpiperidin-1-yl)ethane.

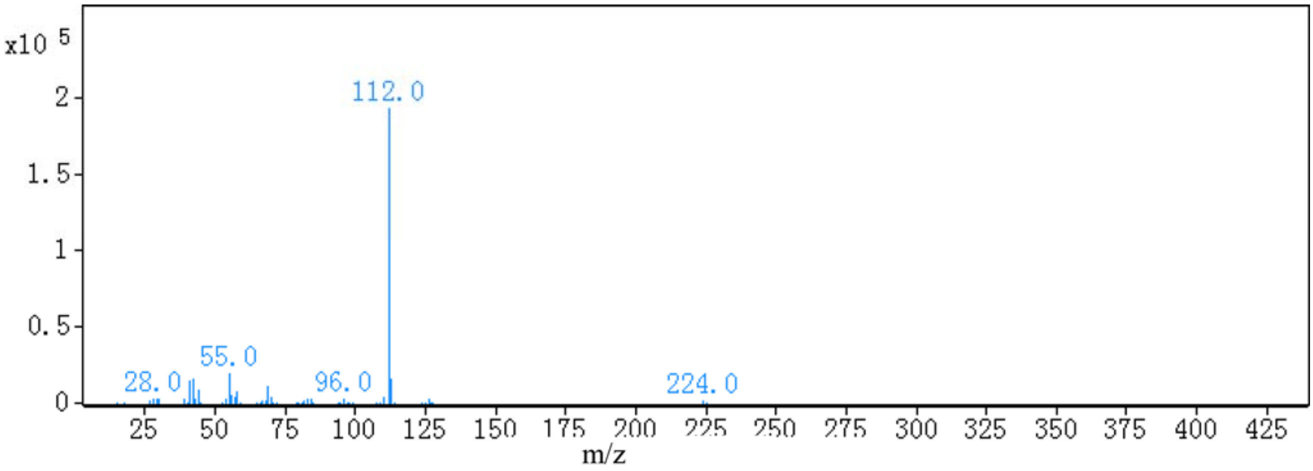

Figure S34. GC-MS spectrum of 1,2-bis(3-methylpiperidin-1-yl)ethane.

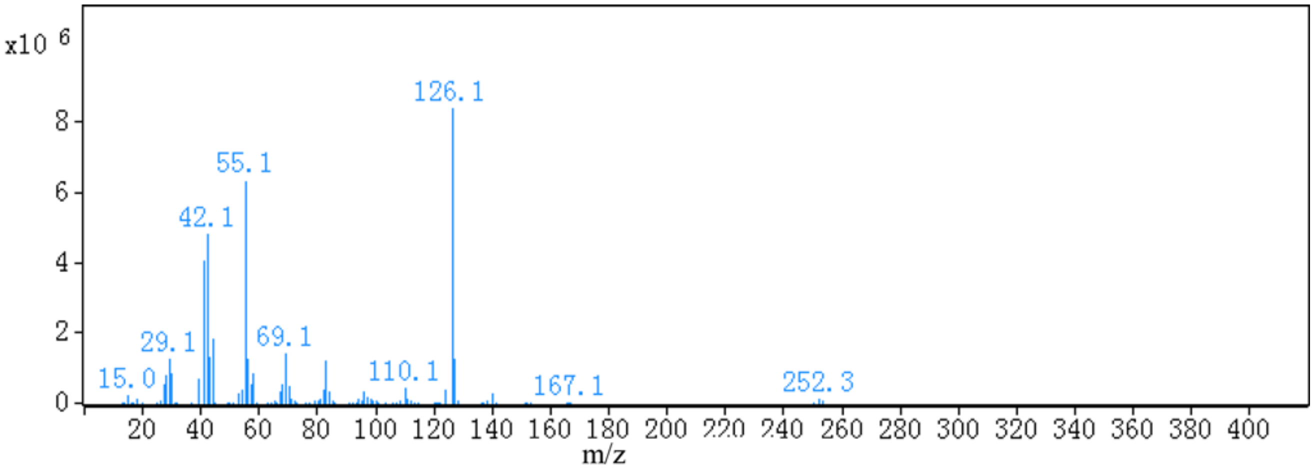

Figure S35. GC-MS spectrum of 1,2-bis(3,5-dimethylpiperidin-1-yl)ethane.

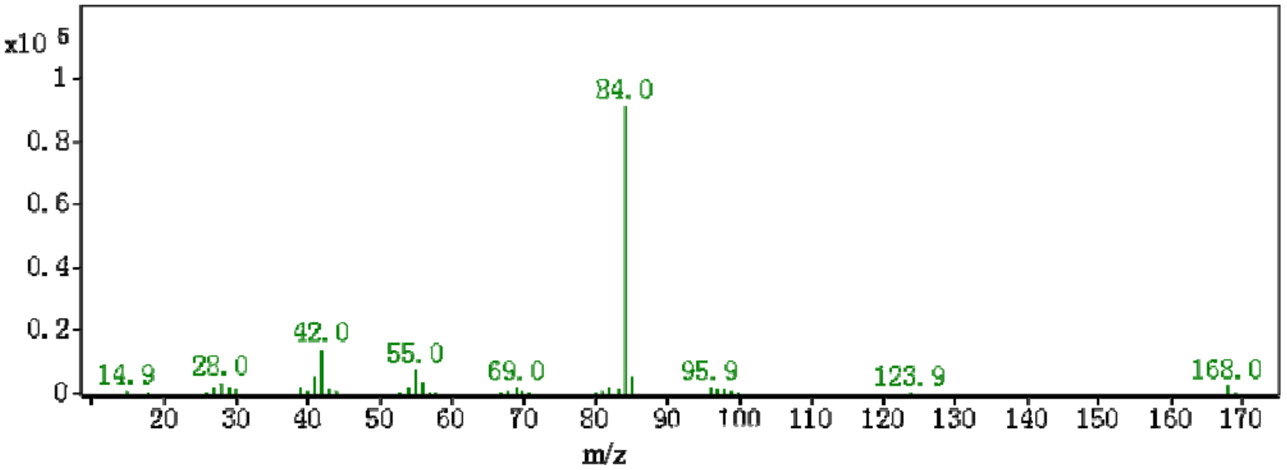

Figure S36. GC-MS spectrum of 1,2-di(pyrrolidin-1-yl)ethane

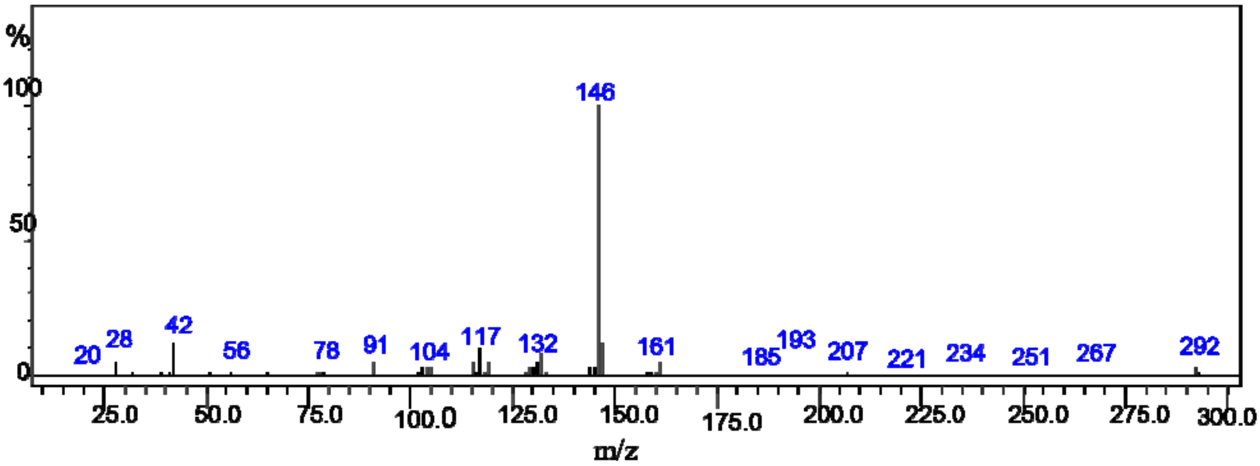

Figure S37. GC-MS spectrum of 1,2-bis-(3,4-dihydro-1H-[2]isoquinolyl)-ethane.

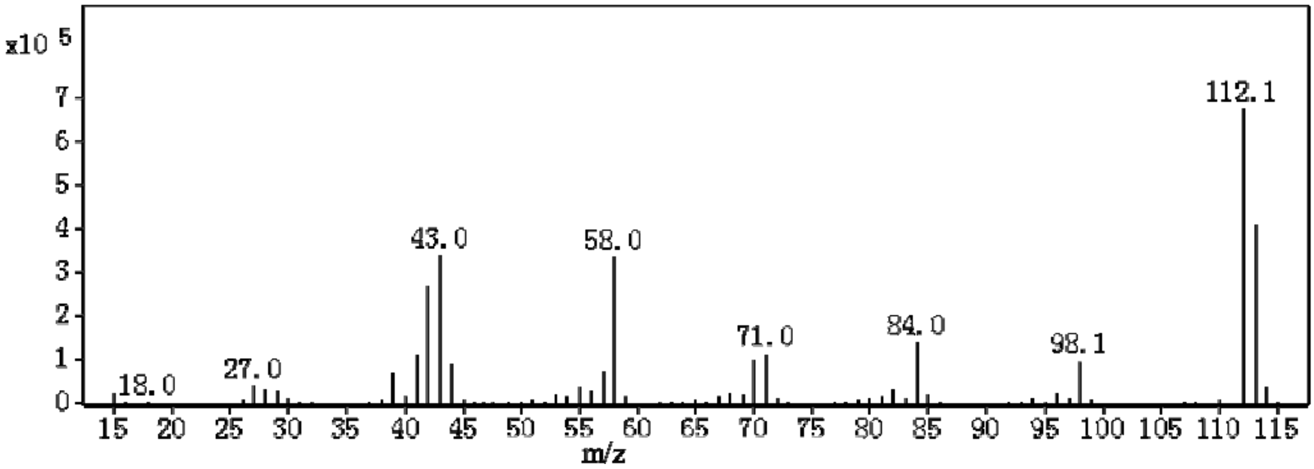

Figure S38. GC-MS spectrum of 1,3-dimethylpiperidine.

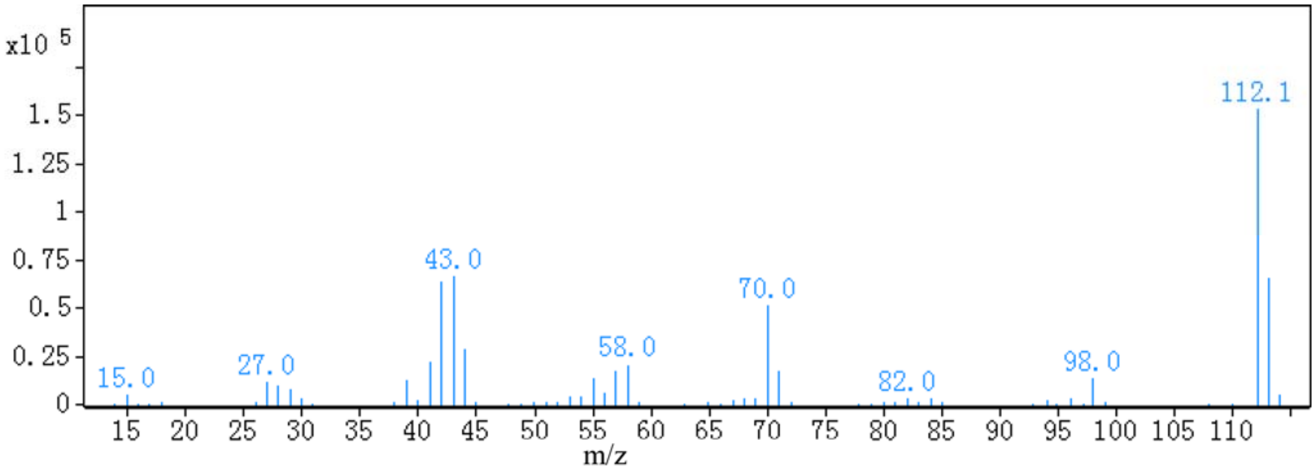

Figure S39. GC-MS spectrum of 1,4-dimethylpiperidine.

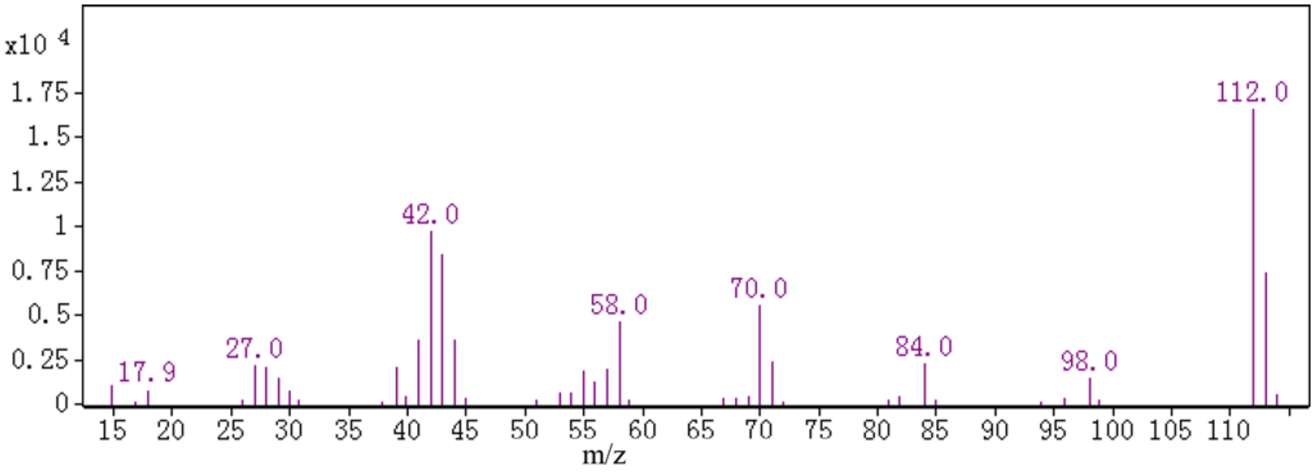

Figure S40. GC-MS spectrum of 1,2-dimethylpiperidine.

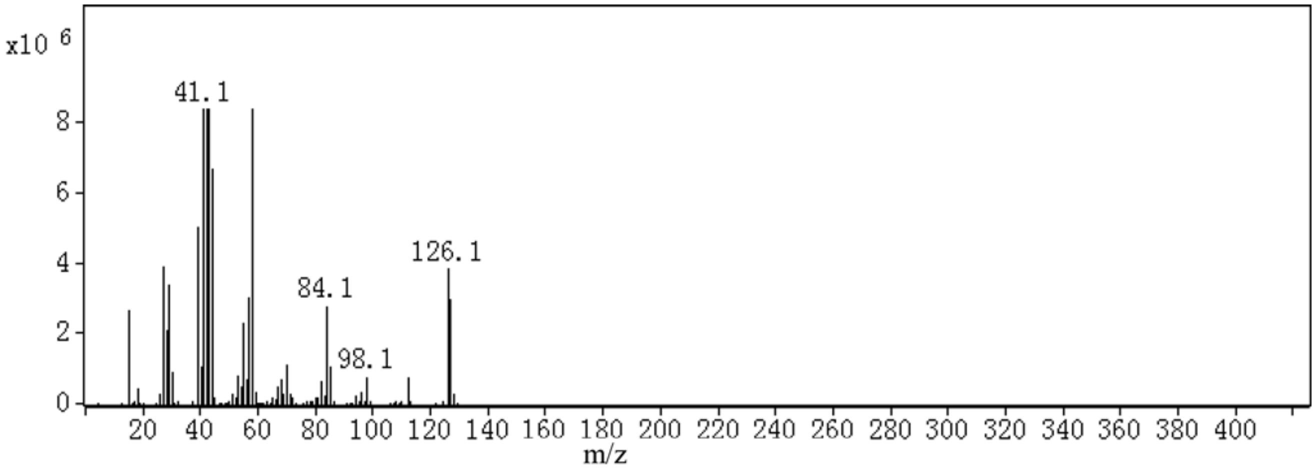

Figure S41. GC-MS spectrum of 1,3,5-trimethylpiperidine.

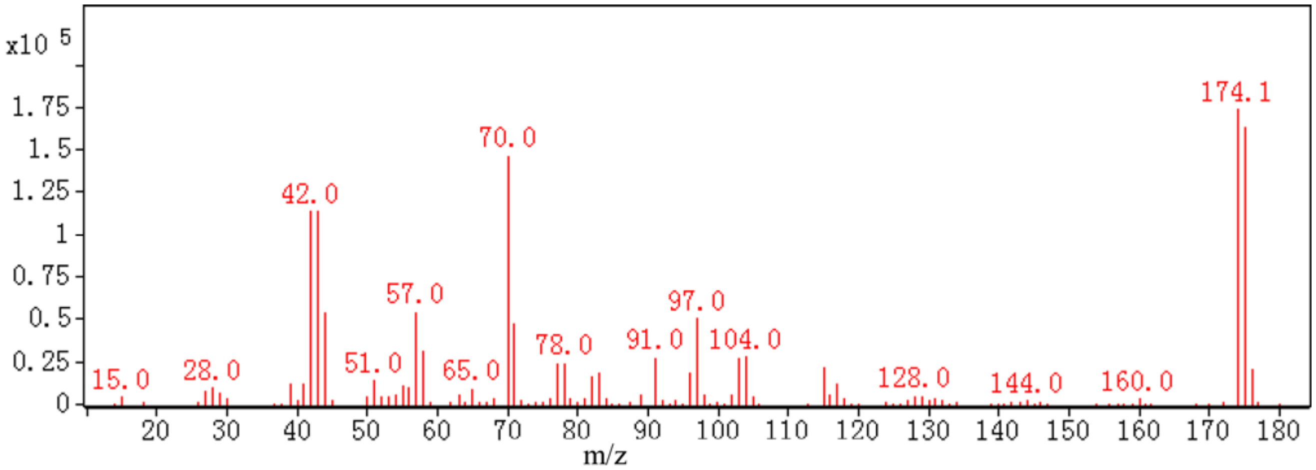

Figure S42. GC-MS spectrum of 1-methyl-4-phenylpiperidine.

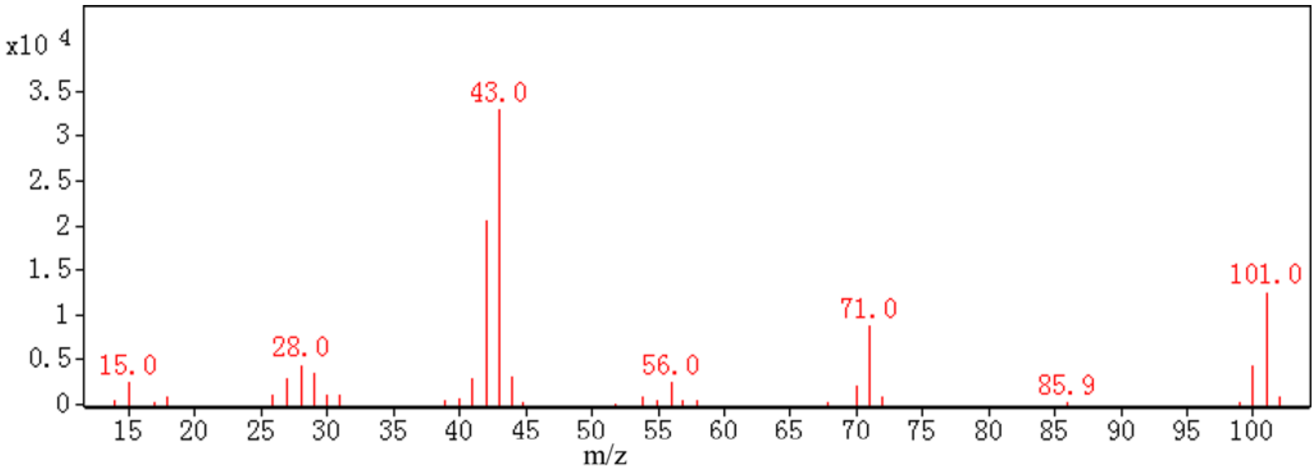

Figure S43. GC-MS spectrum of 4-methyl-morpholine.

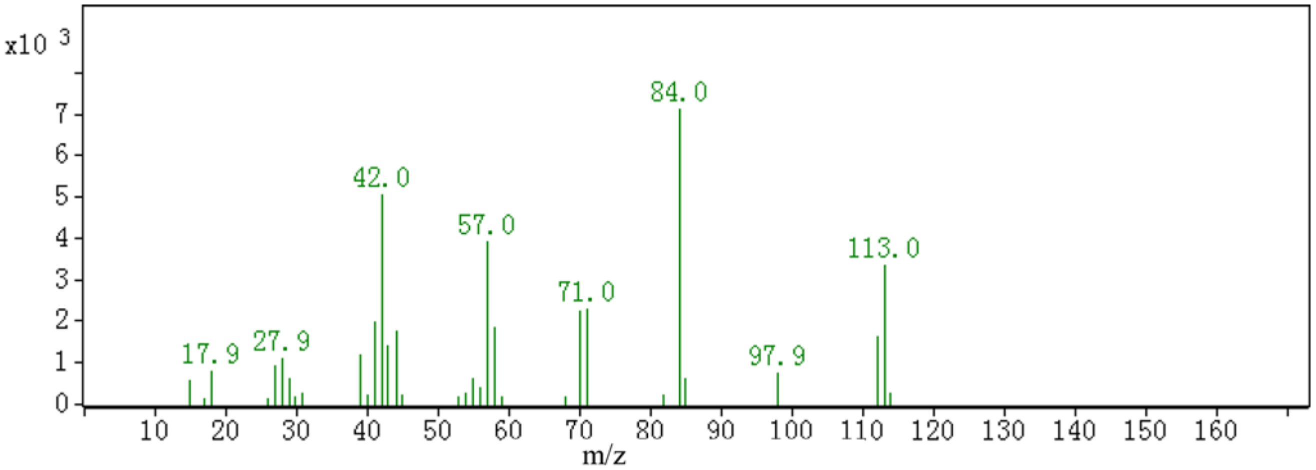

Figure S44. GC-MS spectrum of hexahydro-1-methyl-1H-azepine.

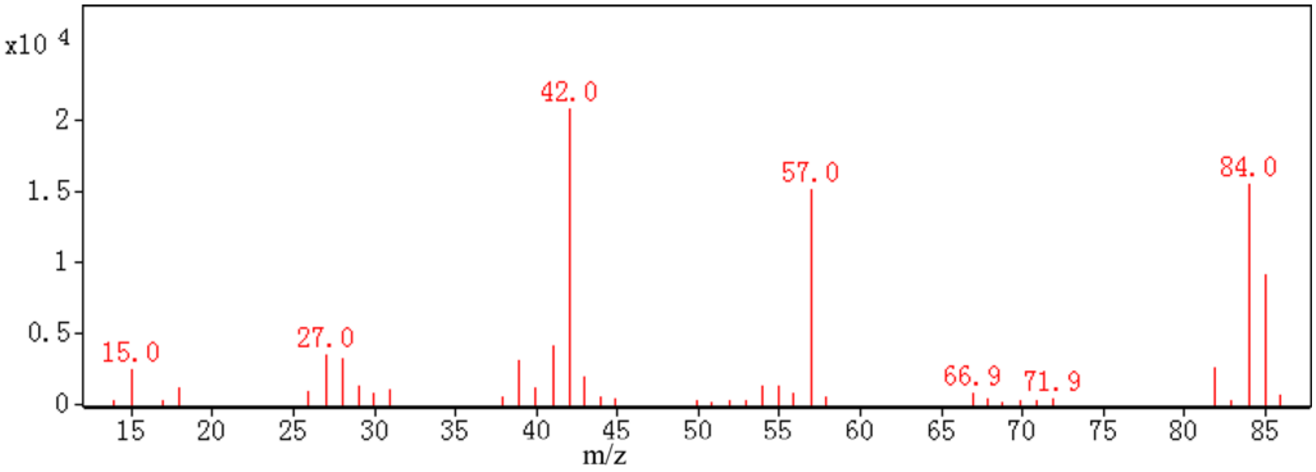

Figure S45. GC-MS spectrum of 1-methylpyrrolidine.

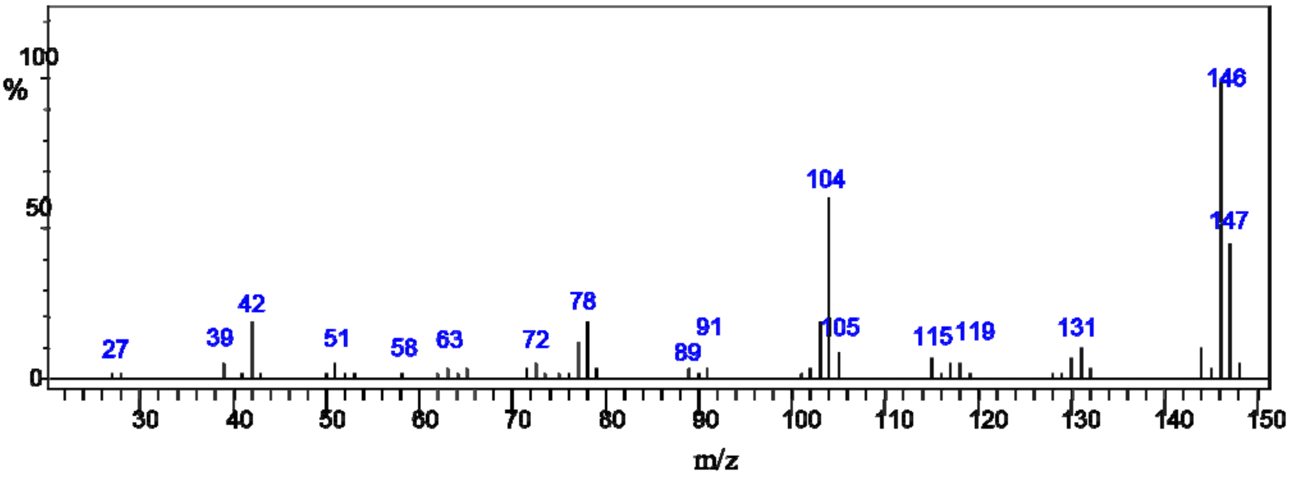

Figure S46. GC-MS spectrum of 2-methyl-1,2,3,4-tetrahydroisoquinoline.

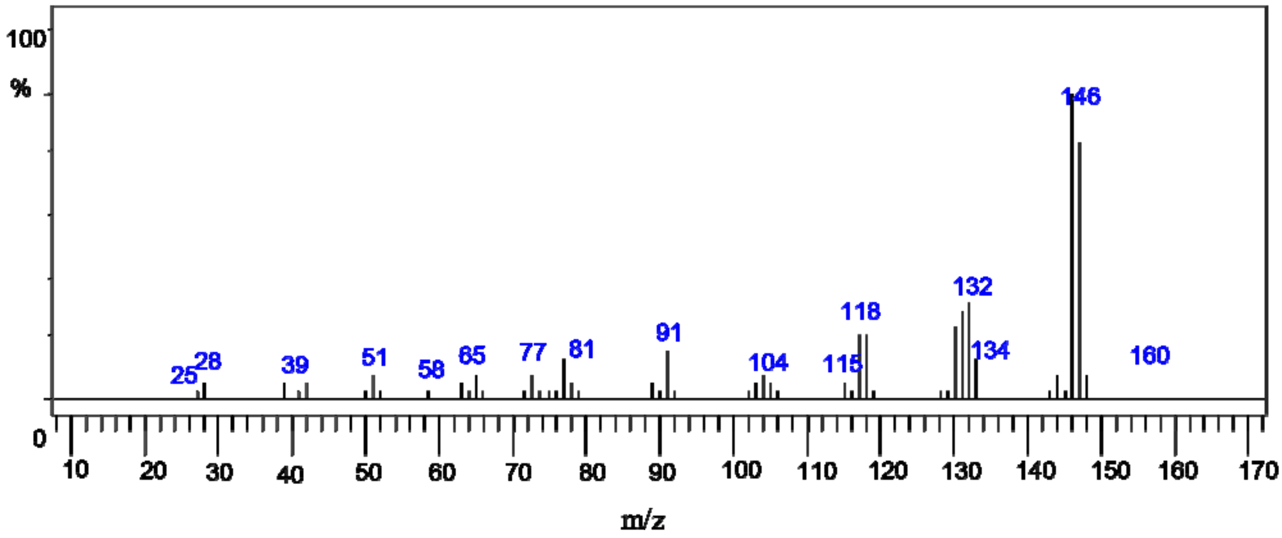

Figure S47. GC-MS spectrum of N-methyl-1,2,3,4-tetrahydroquinoline.
